# Polysaccharides from *Porphyra haitanensis*: A Review of Their Extraction, Modification, Structures, and Bioactivities

**DOI:** 10.3390/molecules29133105

**Published:** 2024-06-29

**Authors:** Minghao Sun, Yuping Zhang, Wuyou Gao, Yujia He, Yu Wang, Yanping Sun, Haixue Kuang

**Affiliations:** Key Laboratory of Basic and Application Research of Beiyao, Heilongjiang University of Chinese Medicine, Ministry of Education, Harbin 150040, China; m13125531353@163.com (M.S.); ping18849676763@126.com (Y.Z.); wuyouwuyou222@163.com (W.G.); heyujia125@163.com (Y.H.); w13504560086@163.com (Y.W.)

**Keywords:** *P. haitanensis* polysaccharides, extraction, structural features, modification, biological activities

## Abstract

*Porphyra haitanensis* (*P. haitanensis*), an important food source for coastal residents in China, has a long history of medicinal and edible value. *P. haitanensis* polysaccharides are some of the main active ingredients in *P. haitanensis*. It is worth noting that *P. haitanensis* polysaccharides have a surprising and satisfactory biological activity, which explains the various benefits of *P. haitanensis* to human health, such as anti-oxidation, immune regulation, anti-allergy, and anticancer properties. Hence, a systematic review aimed at comprehensively summarizing the recent research advances in *P. haitanensis* polysaccharides is necessary for promoting their better understanding. In this review, we systematically and comprehensively summarize the research progress on the extraction, purification, structural characterization, modification, and biological activity of *P. haitanensis* polysaccharides and address the shortcomings of the published research and suggest area of focus for future research, providing a new reference for the exploitation of polysaccharides from *P. haitanensis* in the fields of medicine and functional foods.

## 1. Introduction

*Porphyra haitanensis* (*P. haitanensis*), a temperate plant belonging to the genus *Porphyra* of the family *Bangiaceae*, is mainly distributed along the coasts of the Zhejiang, Fujian, and Guangdong provinces in China [1,2]. *P. haitanensis*, commonly known as Tan-Zi-Cai, is an artificially cultivated seaweed unique to China [3]; it is not only an important food source for coastal residents but also has good medicinal value. According to classic ancient historical books of Chinese medicine, such as “Compendium of Materia Medica” (Ben-Cao-Gang-Ben, the Ming Dynasty) and “Thoroughly Revised Materia Medica” (Ben-Cao-Cong-Xin, the Qing Dynasty), *P. haitanensis* can treat beriberi, edema and cough. In addition, *P. haitanensis* is sweet and salty, cold, and naturally nontoxic and has the ability to resolve phlegm, soften firmness, and clear heat to eliminate toxicity and promote diuresis. Modern research has shown that *P. haitanensis* is rich in proteins, polysaccharides, vitamins, and essential amino acids [4,5,6]. Therefore, *P. haitanensis* has a reputation as a “treasure house of nutrition” and is a high-grade nutritional and health food [7].

Polysaccharides, which are large molecules found in animal cell membranes, as well as in plant and microbial cell walls, are prevalent in biological systems [8,9]. Previous studies have shown that polysaccharides are significant bioactive compounds in *P. haitanensis*, making up approximately 20% to 40% of its composition [10]. *P. haitanensis * polysaccharides are composed primarily of galactose, glucose, mannose, arabinose, fucose, and other monosaccharides. The typical structure of *P. haitanensis* polysaccharides mainly includes repeating units of galactose and 3,6-anhydro galactose, as well as sulfate groups [11,12,13]. In the past two decades, various in vivo and in vitro experimental studies have shown that *P. haitanensis* polysaccharides display various biological activities, including antioxidant [14,15], immunomodulatory [16,17], anti-allergic [18,19], anti-tumor [20,21,22], and anti-aging activities [23,24]. Successive reports of different bioactive polysaccharide compounds extracted from *P. haitanensis* have attracted the interest and attention of an increasing number of pharmacists and chemists. Furthermore, *P. haitanensis* polysaccharides, which are natural and relatively nontoxic active macromolecules, possess medicinal and edible value, making them essential in the realms of healthcare and dietary supplements.

Recently, researchers have extensively studied the extraction, purification, structure, modification, and bioactivity of polysaccharides from *P. haitanensis*. However, there is currently no overview of *P. haitanensis* polysaccharides, which may limit their development and utilization. Based on prior research, this review examines advancements in the extraction, purification, structure, modification, and biological effects of *P. haitanensis* polysaccharides over the past few decades. The main purpose of this study is to fully understand the research progress on *P. haitanensis* polysaccharides and to explore the future development trend of *P. haitanensis* polysaccharides as new drugs and functional foods, to provide new insights and a valuable reference for the further research into and development of *P. haitanensis* polysaccharides.

## 2. Extraction of Polysaccharides from *P. haitanensis*

Extraction is the first indispensable step in the study and utilization of natural polysaccharides [25]. Due to their polar macromolecular nature and intricate structures [26], polysaccharides can undergo glycosidic bond cleavage and conformational changes when subjected to various extraction methods and conditions, ultimately impacting their structure and biological functions [27]. Therefore, scholars have developed and optimized polysaccharide extraction methods to obtain more bioactive polysaccharides from *P. haitanensis*. The known extraction methods for *P. haitanensis*, including hot water extraction (HWE), microwave-assisted extraction (MAE), and ultrasonic–microwave-assisted extraction (UMAE), are shown in Table 1.

**Figure 1:**
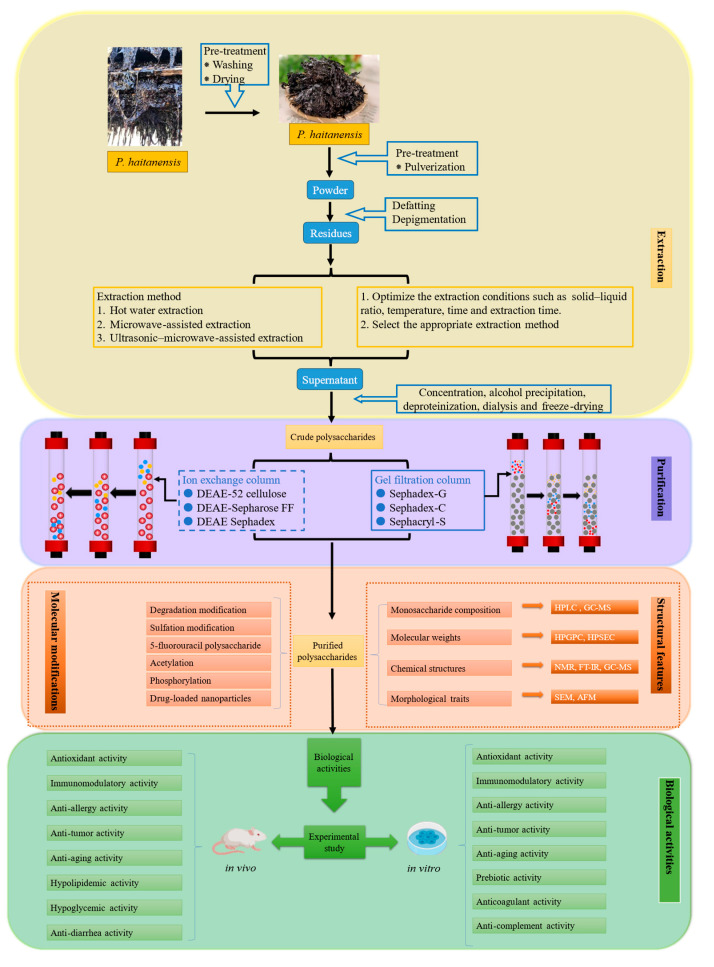
Schematic diagram of extraction, purification, structural features, and biological activities of polysaccharides from *P. haitanensis*.

Generally, to begin the extraction of *P. haitanensis* polysaccharides, a sample of *P. haitanensis* after crushing and drying needs to be soaked in ethanol [13] or treated with methanol/dichloromethane/water for 24 h in a shaking incubator at ambient temperature [11,28] to remove pigments and low-molecular-weight impurities. The detailed extraction and purification steps, structural characteristics, and biological activity of polysaccharides from *P. haitanensis* are shown in Figure 1. According to the literature, the polysaccharide yield of *P. haitanensis* obtained by conventional HWE is generally between 3.3% and 20.33% [11,29,30]. Dong et al. obtained the best extraction conditions for *P. haitanensis* through a Box-Behnken response surface design, in which the ratio of material to water was 0.04, the extraction time was 3 h, and the temperature was 80 °C. This condition had little effect on the structure and function of the polysaccharides, and the extraction rate of PHP-XJ was optimized to 20.33% (±0.15) [31]. Presently, the primary method of extracting *P. haitanensis* polysaccharides is HWE, which is favored for its affordability, ease of use, and low requirement for specialized equipment. The HWE technique can obtain a high yield of polysaccharides via an optimized process, but as a traditional extraction method, HWE still has the disadvantages of a long extraction time, difficult temperature adjustment, and high energy consumption [32].

In recent years, to simplify the extraction conditions of *P. haitanensis* polysaccharides, new extraction methods, such as MAE, have been adopted. By fully consulting the literature, it was found that when the liquid to solid ratio is 30:1–50:1 mL/g, the extraction time is 8–14 min, and the power is 78–300 W, the rate of PHP extraction by the MAE method is 3.6–5.1% [20,32]. The MAE method uses high-frequency electromagnetic waves to penetrate the cell wall, resulting in cell rupture, so that the effective components can be extracted from the medium under uniform heating, eliminating the thermal gradient in the material, thus improving the extraction quality and effectively protecting the functional components in the sample [33,34]. Compared with the conventional HWE method, the MAE method offers benefits such as an adjustable temperature, quick reaction time, and reduced solvent usage [35,36]. Furthermore, Xu et al. used the UMAE method to extract *P. haitanensis* polysaccharides and optimized the extraction method by a response surface methodology. The results showed that the maximum yield of polysaccharides was 20.98% when the temperature was controlled at 80 °C and the ratio of material to liquid was 1:42 g/mL for 30 min [37]. UMAE, as a complementary technology with the combined advantages of MAE and UAE, reduces the extraction temperature, heats the material more evenly, reduces the extraction time, and greatly improves the extraction efficiency.

In summary, the current methods for extracting polysaccharides from *P. haitanensis* are HWE, MAE, and UMAE, but there are few studies on the extraction optimization of the MAE method and UMAE method. In future research, the extraction optimization process of these two methods needs to be further studied. Moreover, although there have been recent developments in polysaccharide extraction technology, we found that there are few methods for polysaccharide extraction from *P. haitanensis*, which undoubtedly limits its research scope. Therefore, new extraction techniques should be investigated in subsequent studies, such as dilute lye and acid extraction [38], enzyme extraction [39], supercritical fluid extraction [40] and ultrahigh-pressure extraction [41]. Regardless of the extraction method, the solvent ratio, temperature, and time affect the relative molecular weight and sulfuric acid content of *P. haitanensis* polysaccharides and subsequently affect their biological activity [8]. Therefore, to increase polysaccharide extraction efficiency, new extraction methods and processes that are highly efficient and obtain high yields with high biological activity need to be explored through scientific research.

**Table 1:** A summary of all the extraction methods of *Porphyra haitanensis* polysaccharides is given in the table below.

**Source**	**Method of Extraction**	**Extraction Times**	**Time (Min)**	**Temperature** **°C**	**Water/Material Ratio** **(mL/g)**		**Yield**	**References**
Xiamen China	HWE	1	240	Boiling	40:1		N/A	[19]
Guangdong China	HWE	1	300	100	50:1		N/A	[42]
Dongtou Zhejiang China	HWE	3	120	95	20:1		N/A	[10]
Pingtan Island Fujian China	HWE	1	120	80	20:1		N/A	[15]
Nan’ao Island Shantou Guangdong China	HWE	1	120	90	30:1		3.3%	[11]
Putian Fujian China	HWE	2	90	80	20:1		3.8%	[29]
Nan’ao Island, Shantou Guangdong China	HWE	1	120	90	30:1		4.10 ± 0.11%	[30]
Zhejiang and Fujian China	HWE	1	118.2	88.4	40:1		15.19%	[28]
China	HWE	1	180	80	25:1		20.33% (±0.15)	[31]
**Source**	**Method of Extraction**	**Extraction times**	**Time (Min)**	**Temperature** **°C**	**Water/Material Ratio (mL/g)**	**Power (W)**	**Yield**	**References**
Hangzhou and Zhejiang China	MAE	1	8	N/A	50:1	300	3.6%	[32]
Jiangsu China	MAE	1	14	N/A	30:1	78	5.01 ± 0.32%	[20]
Nan’ao Island Shantou Guangdong China	UMAE	1	30	80	42:1	500 (Microwave) 50 (Ultrasonic)	20.53%	[37]

N/A means not mentioned.

## 3. Purification of *P. haitanensis* Polysaccharides

The crude polysaccharides obtained by preliminary extraction often contain pigments, inorganic small molecules, proteins, and other impurities, which may affect the analysis and identification of polysaccharides and even cause polysaccharide pollution [43]. Purification is an indispensable part of the study of natural polysaccharides, and its purpose is to purify isolated mixed polysaccharides to obtain various uniform polysaccharides [44]. In particular, highly biologically active nontoxic or low-toxicity components purified from food-borne natural polysaccharides are widely used in food, biological materials, and cosmetics [45]. Therefore, after extracting crude polysaccharides from *P. haitanensis* using the above methods, it is essential to further separate and purify the polysaccharides to obtain high-quality refined polysaccharides to study their structural characteristics and biological activity more accurately. We summarize the steps for separating and purifying *P. haitanensis* polysaccharides in Figure 1, referencing the existing research. First, as mentioned above, *P. haitanensis* is pretreated before extraction to remove pigments and small molecular impurities. Second, the Sevag method [29,32] or Sevag enzymatic (papain) hydrolysis method [13,17] is used to remove macromolecular impure proteins with complex spatial structures, to prevent the combination of proteins and polysaccharides and reduce the difficulty of polysaccharide separation. Then, small molecular impurities (3–10 kDa molecular weight cut-off membrane) are further removed by distilled water dialysis [11,12,15].

Subsequently, the polysaccharides are further separated and purified by gradient elution column chromatography, such as anion exchange chromatography [46], cation exchange chromatography [47], and gel filtration chromatography [48]. *P. haitanensis* polysaccharides are generally purified by anion exchange chromatography and/or gel filtration chromatography, using DEAE-cellulose anion exchange resin [21], DEAE-Sepharose FF anion exchange resin [15], DEAE Sephadex A-50 anion exchange resin [12], Sephadex-G series [7], and Sephacryl-S series gel resin [15], among other resins. Gong et al. used DEAE-cellulose column chromatography to separate and purify crude polysaccharide from *Porphyra haitanensis*. Five polysaccharide components (PP1, PP2, PP3, PP4, and PP5) with antioxidant activity were obtained by elution from 0.1, 0.2, 0.3, 0.6, and 0.7 M aqueous NaCl, respectively [29]. Similarly, Yao et al. obtained *P. haitanensis* polysaccharides by UMAE and DEAE cellulose-52 chromatography, with three components having in vitro anticancer effects [21]. In addition, Zhang et al. applied degraded *P. haitanensis* polysaccharide LP to a DEAE Sephadex A-50 column and gradually eluted it with a stepwise increase in the NaCl concentration to obtain the purified components LP-D1 (yield, 2.15%) and LP-D2 (yield, 31.8%). The purified component LP-D2 was eluted from the Sephadex G-100 column at a flow rate of 0.2 mL/min with tap water. After dialysis and freeze-drying, a galactose sulfate (LP-G2) with anti-complement binding activity was obtained [12]. Subsequently, Chen et al. obtained two purified polysaccharide fractions (PHP0.5 and PHP1.0), accounting for 37.1% and 48.6%, respectively, using a DEAE-Sepharose FF column by stepwise elution with 0.5 and 1.0 mol NaCl solution. The two fractions were eluted from a Sephacryl S-400HR column with distilled water at 1.0 mL/min to obtain PHP0.5–1 and PHP1.0–1 [15].

In summary, high-purity and different kinds of *P. haitanensis* polysaccharides can be obtained by the above methods. However, different column chromatography methods are used in the purification process, and the biological activities of the purified components are also different. In addition, the loss of polysaccharides will inevitably occur during the purification process, and the cost of column chromatography is high, which is challenging for the subsequent development of *P. haitanensis* polysaccharides in functional foods and pharmaceutical products. Therefore, in future research, on the basis of ensuring the rich activity of *P. haitanensis* polysaccharides, green, efficient and economical purification methods for *P. haitanensis* polysaccharides that can be used in industrial production should be sought and optimized to further accelerate the application of *P. haitanensis* polysaccharides in practical markets and increase their use in functional foods and medicine.

## 4. Physicochemical and Structural Characteristics of Polysaccharides from *P. haitanensis*

In recent decades, studies on the physicochemical and structural characteristics of *P. haitanensis* polysaccharides have focused mainly on their monosaccharide composition, relative molecular weight, monosaccharide sequence, chemical structure, type of glycosidic bond, and configuration of glycosidic bonds. The relative molecular mass of *P. haitanensis* polysaccharides is determined by high-performance size exclusion chromatography (HPSEC) and high-performance gel permeation chromatography (HPGPC), while the monosaccharide composition is generally analyzed by gas chromatography-mass spectrometry (GC-MS) or high-performance liquid chromatography (HPLC). The basic chemical structure characteristics of the purified polysaccharide products are further identified by a series of analytical methods, such as gas chromatography (GC), Fourier transform infrared spectroscopy (FT-IR), and nuclear magnetic resonance (NMR). The primary structural characteristics of *P. haitanensis* polysaccharides, including their sulfate content, monosaccharide composition, molecular weight, and chemical structure, are listed in Table 2.

### 4.1. Sulfate Content

Due to the special environment of the ocean, polysaccharides extracted from marine organisms often contain more sulfate groups than those extracted from terrestrial animals, plants, and microbial polysaccharides [49]. Among seaweed plants, red algae are the main source of sulfate polysaccharides. Thus, as a red algae plant, *P. haitanensis* generally contains sulfate galactose polysaccharides [50]. The sulfate content of polysaccharides is generally determined by the BaCl_2_-gelatine turbidimetric technique [11,19]. In the polysaccharides of *P. haitanensis,* sulfate replaces the hydroxyl group on galactose on the polysaccharide chain, mainly in the form of →4)-*α*-L-galactose-6-sulfate linkages [12]. Research has shown that sulfate accounts for a large proportion of polysaccharide components in *P. haitanensis* and that the sulfate content of various *P. haitanensis* polysaccharides generally ranges from 3.7–14.7%, as shown in Table 2. In addition, Ji et al. investigated the effects of different production areas and harvesting periods on the sulfate content in polysaccharides from *P. haitanensis.* The results showed that the polysaccharide PHPR03 extracted from *Porphyra haitanensis* picked in the third harvest period (late December) in Raoping had the highest sulfate content (14.70%), indicating that the sulfate content of *P. haitanensis* polysaccharides was also affected by the production area and harvesting period [51]. Recent research indicates that the sulfate content is closely linked to the biological activity of polysaccharides derived from *P. haitanensis*. For example, Wang et al. reported that as the harvest period increased, the sulfate content in *P. haitanensis* polysaccharides decreased gradually, while the anti-allergic properties increased gradually [13]. Moreover, Wu et al. reported that among the three polysaccharides isolated from *P. haitanensis*, the purified component PHP3 eluted with 0.7 M NaCl on a Sephadex G-100 column had the highest level of sulfate and the most potent antioxidant properties, suggesting that as the sulfate content increases, so does the antioxidant activity of polysaccharides [14]. In summary, the sulfate group, as an important component of *P. haitanensis* polysaccharides, affects the structure and activity of polysaccharides. In the future, it will be highly important to isolate new active sulfated *P. haitanensis* polysaccharides for development and utilization.

### 4.2. Monosaccharide Composition

Generally, the monosaccharide composition of *P. haitanensis* polysaccharides is analyzed by acid hydrolysis and derivatization, followed by detection via GC-MS and HPLC. According to the published studies, a significant portion of the monosaccharide composition of polysaccharides from *P. haitanensis* is galactose. The galactose content of *P. haitanensis* polysaccharide obtained via different methods generally accounts for 24.35% to 95.60% of its monosaccharide composition. Chen et al. separated and purified water-extracted PHPs using a DEAE-Sepharose FF column and Sephacryl S-400HR column and obtained the homogeneous polysaccharide, PHP1, with only galactose in its monosaccharide composition [52]. Furthermore, the extraction, separation, and purification techniques for *P. haitanensis* vary depending on the source, resulting in differing types and ratios of monosaccharides in the polysaccharides, leading to varying biological activities. For example, through water extraction, a probiotic active polysaccharide from *P. haitanensis*, known as PHP, was acquired. The composition of the substance was primarily galactose, glucose, and mannose, determined through high-performance liquid chromatography, with a molar ratio of 94.85:3.18:1.97 [30]. Moreover, Wang et al. further purified *P. haitanensis* polysaccharides by DEAEseult52 column chromatography and Sephadex G-100 gel column chromatography to isolate a polysaccharide, PH, with anti-colon cancer activity. GC-MS analysis revealed that PH was composed of Gal, Glu, Man, Ara, Fuc, Xyl, and Rha in a molar ratio of 98.66:0.23:0.49:0.07:0.05:0.07:0.44 [22]. Three antioxidant polysaccharides, PHP1, PHP2, and PHP3, were obtained from PHPs by cellulose DEAE-52 and Sephadex G-100 column chromatography. GC-MS analysis revealed that these polysaccharides contained Rha, Xyl, Gal, Glc, and Man. Among them, the proportions of Rha and Xyl in PHP3 were greater than those in the other two polysaccharides, and the antioxidant activity of PHP3 was also the strongest, suggesting that the presence of Rha and Xyl in polysaccharides from *P. haitanensis* may affect their antioxidant properties [14]. In addition to the above monosaccharides, *P. haitanensis* polysaccharides also contain 3,6-anhydrogalactose (3,6-AG), a crucial bioactive compound found in red algae polysaccharides [30]. In the study of *P. haitanensis* polysaccharides, some researchers have regarded 3,6-AG as a separate component different from the composition of monosaccharides [19], but others classify 3,6-AG as a monosaccharide [29]. However, regardless of the type of division, 3,6-AG is an important component of *P. haitanensis* polysaccharides. Furthermore, the variation in 3,6-AG levels and monosaccharide makeup in *P. haitanensis* polysaccharides is likely due to the source and timing of harvest of the raw materials, as well as the methods used for extraction and purification [32]. Table 2 contains a comprehensive breakdown of the monosaccharide compositions found in the polysaccharides of *P. haitanensis*.

In summary, the different types and proportions of monosaccharides in the monosaccharide composition of *P. haitanensis* polysaccharides potentially affect their bioactivities. Hence, in future research, a more efficient and accurate ion chromatography method is needed to accurately determine the monosaccharide composition of *P. haitanensis* polysaccharides, laying a reliable foundation for subsequent research. In addition, for the diversification of the monosaccharide composition of *P. haitanensis* polysaccharides from different sources and different harvests, the fingerprint of their monosaccharide composition should be established in subsequent studies, and a reasonable quality evaluation, as well as biological activity assessment, of *P. haitanensis* polysaccharides should be carried out.

### 4.3. Molecular Weight

The average molecular weight (Mw), as a vital parameter for chemical compounds, is closely related to the structure and functional biological activity of polysaccharides. The molecular weight of *P. haitanensis* polysaccharides is analyzed mainly by HPGPC [12,13,14,53] and HPSEC [11]. However, even for the same polysaccharide, the Mw detection results are different due to different extraction and purification processes and analysis methods. For example, the Mw of PHPs extracted from hot water was measured to be 201 kDa by HPSEC [11]; interestingly, for PHPs extracted from *P. haitanensis* using the same extraction method and analyzed by HPSEC-RID, their molecular weight was 250 kDa [54]. In addition, Wu et al. used HPGPC to determine molecular weights, obtaining three polysaccharides, namely, PHP1, PHP2, and PHP3, with relative molecular weights of 499, 523, and 797 kDa, respectively, by DEAE-cellulose column separation and the purification of PHPs [14]. Additionally, Wang et al. extracted and purified *P. haitanensis* from different harvest periods in November, December, and January and obtained three polysaccharides, namely, PHP1 (November), PHP2 (December), and PHP3 (January), with each exhibiting anti-allergic activity; the molecular weights were 567.05, 414.09, and 323.80 kDa, respectively. Notably, with the extension of the harvest period, the relative molecular mass of PHPS gradually decreased, but the anti-allergic activity gradually increased [13]. The process, separation method, and detection conditions affect the molecular weight of polysaccharides, and the *P. haitanensis* itself (such as its source or harvest period) has a significant effect on the molecular weight, which in turn affects the biological activity of the polysaccharides.

### 4.4. Chemical Structure

**Figure 2:**
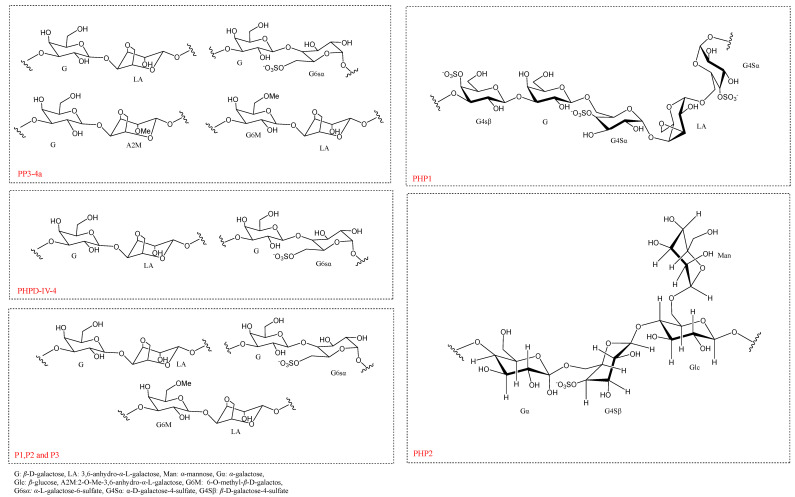
The hypothetical structure of *P. haitanensis* polysaccharides [7,52,53,57].

In recent years, researchers have extracted a variety of polysaccharides with different structural characteristics from *P. haitanensis*, and their chemical structures have been analyzed by IR spectroscopy, NMR spectroscopy, and GC-MS. However, compared with studies of monosaccharide composition and relative molecular mass, there are few studies on the chemical structure and conformational characteristics of *P. haitanensis* polysaccharides. Therefore, we summarize and analyze the structural characteristics of *P. haitanensis* polysaccharides, referencing published studies to provide valuable information for subsequent research. In fact, as early as 1993, using NMR spectroscopy, scholars verified that the main repeating unit in polysaccharides of *P. haitanensis* is (1→3-*β*-D-galactopyranosyl-(1→4)-6-sulfate or -L-galactopyranose [55]. According to FT-IR and NMR results, the sulfated galactan in *P. haitanensis* has a typical linear skeleton of 4-linked *α*-L-galactosyl 6-sulfate and 3,6-anhydro-*α*-L-galactosyl units and 3-linked *β*-D-galactosyl units [56]. Similarly, through the analysis of 1D/2D NMR and FT-IR spectroscopy data, it was speculated that the structure of a degraded *P. haitanensis* polysaccharide (PP3-4a) was mostly composed of two →3)*β*-D-galactose (1→4) 3,6-anhydro-*α*-L-galactose (1→, and →3) *β*-D-galactose (1→4) *α*-D-galactose-6-S (1→ repeating structural units), and the methoxy group replaced the C-2 position of 3,6-anhydrogalactose and the C-6 position of galactose [29]. Furthermore, Gong et al. analyzed the structure of a purified degraded polysaccharide, PHPD-IV-4, by 1D/2D-NMR and monosaccharide composition analysis; interestingly, its structure is also a repeating unit of →3) *β*-D-galactose (1→4) 3,6-anhydro-*α*-L-galactose (1→, and →3) *β*-D-galactose (1→4) *α*-L-galactose-6-S (1→ [7]. According to the above description, the structure of degraded *P. haitanensis* polysaccharides is mainly composed of two parts. For example, the main structural components of PHPD-IV-4 are shown in Figure 2 as G-L6S and G-A [7]. On this basis, Hu et al. found that in addition to the typical repeat unit structures G-A and G-L6S, the structures of the three degraded polysaccharides (P1, P2, and P3) purified and isolated by a DEAE cellulose-52 column and Sephadex G-100 column also contained →3)6-O-methyl-*β*-D-galactose (1→4) 3,6-anhydro-*α*-L-galactose (1→ [53], as shown in Figure 2. Moreover, an anti-complementary sulfated heterogalactan, degraded polysaccharide (LP-G2), was isolated from *P. haitanensis*. The backbone of LP-G2, according to NMR spectroscopy analysis, consisted of →4)-*β*-D-galactose→4)-*α*-L-galactose-6 sulfate segments, with *β*-D-Glc and the *α*-D-galactose unit substituted at the 6-position of *α*-L-galactose [12]. Similarly, Khan et al. determined the structure of →4-3,6-anhydro-*α*-L-galactopyranose-(1→3)-*β*-D-galactopyranose segments in *P. haitanensis* crude polysaccharide (PHP-KC-AC) by FT-IR and 1D/2D NMR [11]. Moreover, Chen et al. isolated two purified polysaccharides, PHP1 and PHP2, from *P. haitanensis* polysaccharides (PHPs). By combining GC-FID, GC-MS, FT-IR, and NMR analyses, the structure of the sulfated polysaccharide PHP1 was identified as →3) G4S*β* (1→3) G (1→6) G4S*α* (1→4) LA (1→6) G4S*α* (1→ [52]. Similarly, Chen et al. proposed a hypothetical structure of PHP2 by combining infrared spectroscopy, methylation analysis, and nuclear magnetic resonance spectroscopy data. This structure comprises →4) G*α* (1→6) G4S*β* (1→4) Glc (1→, with a side chain of Man (1→6) Glc [57], as illustrated in Figure 2. The above results show that the different *P. haitanensis* polysaccharides and their derivatives have similar carbon chain skeletons, but that due to the different extraction and purification processes and analysis methods, their monosaccharide categories, glycosidic bond linkages, and conformations may be different. At present, there are few studies on the chemical structure and conformation of *P. haitanensis* polysaccharides, which undoubtedly increases the difficulty of accurate identification of the structure of *P. haitanensis* polysaccharides and subsequent product development. Therefore, we should further investigate the structure of *P. haitanensis* polysaccharides, to further explore the relationship between the biological activity of *P. haitanensis* polysaccharides and their complex spatial structure and provide a basic structural theory for the development and utilization of *P. haitanensis* polysaccharides.

### 4.5. Morphological Traits

The surface microstructure is also an important parameter for analyzing polysaccharides and is helpful for observing the diversity of polysaccharide structures and verifying the relationship between chemical groups and the conformation of polysaccharides. In recent years, the molecular morphology of polysaccharides from *P. haitanensis* has been analyzed by atomic force microscopy (AFM) and scanning electron microscopy (SEM). For example, the microstructure of PHSP_(hp)_ was found to have an irregular geometric shape, a gap on the surface, and a loose overlap under SEM [58]. In addition, Khan used SEM technology to determine that PHP had hexagons and a small number of rectangular pores, in addition to an irregular network structure and a loose microstructure. This loose and highly porous structure had good water absorption and water retention. Additionally, AFM once again confirmed the hollow structure of PHP-KC-AC. Under an atomic force microscope, some irregular fiber networks with hexagonal network structures can be observed, and the fiber height ranges from 0.23 to 0.92 nm, with an average of 0.45 ± 0.17 nm [11]. Moreover, by using SEM and AFM, Ji et al. reported that PHPR03 exhibited a high level of antioxidant activity due to its loose microstructure, strong aggregation, and branching structure [51]. The loose and dense porous structure of PHP03 suggested that it possesses excellent hydration properties, allowing complete absorption by the human body to enhance its antioxidant effects. Chen et al. analyzed two purified sulfated polysaccharides, PHP1 and PHP2, by AFM. The 3D AFM image of PHP1 showed a relief map of the aggregated polysaccharide chains, indicating that there was a strong interaction between the polysaccharide chains [52]. In addition, as viewed with nano-analysis software, PHP2 exhibited a nonuniform spherical chain structure, indicating that the aggregates were significantly wider and longer than the individual polysaccharide chains [57]. From the above microstructure, it can be inferred that the presence of sulfate groups between PHP1 and PHP2 can cause distortion and mutual transformation between sugar ring conformations and that hydroxyl groups can also interact with the branched chain structure. In short, the study and analysis of the surface ultrastructure characteristics of *P. haitanensis* polysaccharides can aid in understanding their chemical structure and spatial conformation, and the microstructural characteristics of polysaccharides can also reflect their physical and chemical properties and be used to identify potential biological activities. Therefore, in future development, the surface characteristics of the polysaccharides of *P. haitanensis* should be modified so that they can be used as bioactive materials, thus expanding the research and development scope of *P. haitanensis*.

**Table 2:** Monosaccharide composition and chemical structure of polysaccharides from *Porphyra haitanensis*.

No.	Polysaccharide Names	Molecular Weight (Da)	Sulfate Conent (%)	Monosaccharide Composition	Structural Characterization	Analysis Technique	References
1	PHP-KC	2.01 × 10^5^	12.61 ± 0.44%	Gal, Glu, and Man in a molar ratio of 94.85:3.18:1.97	(1→4)-linked 3,6-anhydro-*α*-L-galactopyranose units or (1→4)-linked *α*-L-galactose 6 sulphate units	HPSEC-RID, HPLC FT-IR, NMR	[30,54]
2	PHP-KC-AC	2.5 × 10^5^	3.8 ± 0.3%	Gal and 3,6-AG in a molar ratio of 1.2:1.0	→4–3,6-anhydro-*α*-L- galactopyranose (1→3) *β*-D-galactopyranose segments	HPSEC GC-MS, FT-IR, NMR	[11]
3	PP3-4a	2 × 10^4^	19.8%	Gal and 3,6-AG	→3) *β*-D-galactose (1→4) 3,6-anhydro-*α*-L-galactose (1→, and →3) *β*-D- galactose (1→4) *α*-L-galactose-6-S (1→, repeating structural units	HPGPC, GC-MS, NMR	[29]
4	PY1	N/A	5.4%	Gal and a little of Xyl and Ara	*α*-glycosidic bonds	HPLC-GPC,FT-IR, GC	[32]
5	PY2	N/A	3.7%	Gal and a little of Ara, Xyl, Glu, and Man.	*β*-amide pyranose		
6	CPHP-TZ	5.38 × 10^5^	6.48%	Rha, Xyl, Man, Glu, and Gal in a molar ratio of 3.67:2.31:2.49:1:246.64	N/A	HPGPC, GC-MS, FT-IR, UV–Vis, NMR	[14]
7	PHP1-TZ	4.99 × 10^5^	7.11%	Rha, Xyl, Man, Glu, and Gal in a molar ratio of 1.21:4.36:1.36:1:705.86	Contains *α*-type and *β*-type glycosidic bonds		
8	PHP2-TZ	5.23 × 10^5^	8.33%	Rha, Xyl, Man, Glu, and Gal in a molar ratio of 2:3:2.6:1:990.3			
9	PHP3-TZ	7.97 × 10^5^	11.96%	Rha, Xyl, Man, Glu, and Gal in a molar ratio of 9.1:6.3:2.4:1:960.8			
10	PHPD-IV-4	1.0 × 10^4^ (Before purification)	N/A	Gal and 3,6-AG	repeat units of →3) *β*-D-galactose (1→4) 3,6-anhydro-*α*-L-galactose (1→, and→3) *β*-D-galactose (1→4) *α*-L-galactose-6-S (1→.	HPGPC, FT-IR, NMR	[7]
11	PHP1-BZ	5.46 × 10^5^ g/mol	6.93 ± 0.05%	Gal	→3) *β*-D-galactose-4-sulfate (1→3) *β*-D-galactose (1→6) *α*-D-galactose-4-sulfate (1→4) 3,6-anhydro-*α*-L-galactose (→6) *α*-D-galactose-4-sulfate (1→	GC-FID, GC-MS, FT-IR, NMR	[52]
12	PHP2-BZ	1.14 × 10^6^ (±3.44%) g/mol	5.07 ± 0.04%	Gala, Man, and Glu in a molar ratio of 69.27:21.32:9.41	→4) *α*-galactose (1→6) *β*-D-galactose-4-sulfate (1→4) *β*-glucose (1→ and a side chain of *α*-mannose (1→6) β-glucose	GC, FT-IR, NMR, GC-MS	[57]
13	LP-G2	8381	12.94%	Gal, GalA, Glu, and Ara in a molar ratio of 10.46:14.10:0.33:1.52:0.04	→4) *β*-D-galactose→4) *α*-L-galactose-6-sulfate segments, with β-D-glucose and *α*-D-galactose unit substituted at the 6-position of *α*-L-galactose.	HP-GPC, HPLC, NMR, IR, GC-MS.	[12]
14	P1	3.003 × 10^5^	8.42%	Gal, Glu, Man, Ara, Rha, Xyl, and Fuc in a molar ratio of 97.48:1.31:0.33:0.39:0.22:0.18:0.10	repeat units of→3) *β*-D-galactose (1→4) 3,6-anhydro-*α*-L-galactose (1→, and→3) *β*-D-galactose (1→4) *α*-L-galactose-6-S (1→, and→3) 6-O-methyl-*β*-D-galactose (1→4) 3,6-anhydro-*α*-L- galactose (1→.	HPGPC, GC-MS, UV, NMR	[53]
15	P2	1.304 × 10^5^	9.48%	Gal, Glu, Man, Ara, Rha, Xyl, and Fuc in a molar ratio of 97.1:1.18:1.39:0.18:0.07:0.06:0.03			
16	P3	1.115 × 10^5^	13.68%	Gal, Glu, Man, Ara, Rha, Xyl, and Fuc in a molar ratio of 98.62:0.73:0.24:0.12:0.21:0.04:0.04			
17	PHPW	N/A	12.79 ± 1.20%	GulUA, Man, Rib, Rha, GlcN, GalUA, GalN, Glc, Gal, Ara, and Fuc in a molar ratio of 0.01:0.33:0.34:0.04:0.08:0.21:0.44:0.65:33.19:0.07:3.27	N/A	HPLC, GPC FTIR	[51]
18	PHPX	N/A	8.00 ± 0.75%	GulUA, ManUA, Man, Rib, Rha, GlcN, GalUA, GalN, Glc, Gal, Ara, and Fuc in a molar ratio of 0.02:0.04:0.31:0.49:0.11:0.10:0.27:1.35:0.65:34.17:0.02:2.85	N/A		
19	PHPZ	N/A	9.28 ± 0.15%	GulUA, Man, Rib, Rha, GlcN, GalUA, GalN, Glc, Gal, Ara, and Fuc in a molar ratio of 0.02:0.49:0.64:0.04:0.02:0.09:0.30:0.18:37.22:0.02:3.70	N/A		
20	PHPR	N/A	14.38 ± 0.45%	GulUA, Man, Rib, GlcN, GalUA, GalN, Glc, Gal, and Fuc in a molar ratio of 0.05:0.11:0.28:0.04:0.07:0.47:0.62:24.39:4.4	N/A		
21	PHPN	N/A	13.11 ± 1.05%	GulUA, Man, Rib, GlcN, GalUA, GalN, Glc, Gal, and Fuc in a molar ratio of 0.04:0.28:0.44:0.02:0.06:1.12:34.35:4.85	N/A		
22	PHPR01	N/A	10.45 ± 0.90%	Man, Rib, Rha, GlcN, GalUA, GalN, Glc, Gal, Ara, and Fuc in a molar ratio of 0.16:0.42:0.01:0.09:0.15:2.00:0.69:33.98:0.07:5.22	N/A		
23	PHPR02	N/A	13.75 ± 1.65%	Man, Rib, GlcN, GalUA, GalN, Glc, Gal, and Fuc in a molar ratio of 0.27:0.52:0.01:0.03:2.06:0.18:35.05:6.11	N/A		
24	PHPR03	6.7 × 10^5^	14.70 ± 2.11%	Man, Rib, Rha, GlcN, GalUA, GalN, Glc, Gal, Ara, and Fuc in a molar ratio of 0.22:0.50:0.04:0.05:1.62:0.19:33.78:0.04:6.70	N/A		
25	PHPR04	N/A	12.90 ± 0.45%	Man, Rib., Glu, GalUA, Gal, Glu, Gal, Ara, and Fuc in a molar ratio of 0.10:0.41:0.02:0.04:2.45:0.14:29.88:0.03:7.55	N/A		
26	PHPR05	N/A	6.72 ± 0.15%	Man, Rib., Glu, GalUA, Gal, Glu, Gal, Ara, and Fuc in a molar ratio of 0.16:0.36:0.05:0.11:0.26:0.45:35.34:0.03:4.16	N/A		
27	PHP1-LF	5.6705 × 10^5^	8.36 ± 0.16%	Gal, Glc, Xyl, Man, Fru, and Glc-UA in a molar ratio of 98.10:0.54:0.19:0.36:0.15:0.66	N/A	HPGPC, IC FT-IR UV-vis	[13]
28	PHP2-LF	4.1409 × 10^5^	7.53 ± 0.53%	Gal, Glc, Xyl, Man, Fru, and Glc-UA in a molar ratio of 94.27:3.95:0.28:0.46:0.26:0.78	N/A		
29	PHP3-LF	3.2380 × 10^5^	4.23 ± 0.59%	Gal, Glc, Xyl, Man, Fru, and Glc-UA in a molar ratio of 96.91:1.66:0.19:0.54:0.17:0.53	N/A		
30	PH	5.23 × 10^5^	5.28%	Gal, Glu, Man, Ara, Fuc, Xyl, and Rha in a molar ratio of 98.66:0.23:0.49:0.07:0.05:0.07:0.44	N/A	HPLC GC-MS	[22]
31	CPH-TZ-AO	5.24 × 10^5^	8.42%	Gal, Glc, Man, Ara, Fuc, Xyl, and Rha in a molar ratio of 98.66:0.23:0.49:0.07:0.05:0.07:0.44	N/A	HPGPC GC-MS FT-IR UV	[17]
32	DCHP-TZ-AO	2.17 × 10^5^	13.68%	Gal, Glc, Man, Ara, Fuc, Xyl, and Rha in a molar ratio of 95.60:2.01:1.34:0.20:0.08:0.09:0.70	N/A		
33	PHP-XJ	6.3 × 10^5^	2.7 mg/mL	Glu, Gal, and Fuc in a molar ratio of 2.1:76.2:1	N/A	HPGPC LC	[31]

## 5. Molecular Modifications of Polysaccharides from *P. haitanensis*

Polysaccharides, diverse large biological molecules, exhibit biological functions that are intricately linked to their structural composition [59]. The methods used to extract and modify polysaccharides directly affect their composition and biological properties. The chemical modification of polysaccharides can significantly increase structural diversity, promote biological activity, and even increase new biological activity [60]. *P. haitanensis* polysaccharides have rich functional groups and a variety of bioactivities. Molecular modification may improve the biological activity of *P. haitanensis* polysaccharides by changing their chemical structure. Common methods include polysaccharide degradation, phosphorylation, sulfation, acetylation, and the creation of drug-loaded nanoparticles. Previous studies have confirmed that chemical modification can effectively improve the structural and functional properties of polysaccharides from *Porphyra haitanensis*. Table 3 lists the modifications of *P. haitanensis* polysaccharides.

### 5.1. Degradation Modification

Degradation modification can reduce the molecular weight of polysaccharides and enhance their water solubility, so as to solve the problem that polysaccharides with too large a molecular weight have difficulty entering cells and cannot exert their activity [61]. Currently, the methods used to degrade polysaccharides in *P. haitanensis* include the ascorbate (vitamin C) and hydrogen peroxide methods [7,29,62] and the pectinase degradation method [17], among which the ascorbate and hydrogen peroxide methods are the most common. Based on the published studies, we found that degraded polysaccharides of *P. haitanensis* display rich biological activities, such as antioxidation [29,62], immune regulation [7,17,53], anti-allergy [18] and antidepressant [63] properties, neurotoxin inhibition, and memory improvement [64]. Furthermore, studies have shown that the activity of the degraded polysaccharide is different from that of the original polysaccharide. For example, after treatment with ascorbate and hydrogen peroxide, degraded polysaccharides of *P. haitanensis* have a greater ability to reduce iron ions and eliminate free radicals than do the original polysaccharide, showing stronger antioxidant activity [53,62]. Similarly, Gong et al. reacted the purified polysaccharide PP3 with different proportions of H_2_O_2_-VC for different durations and obtained polysaccharides with different Mw values. The ability of the degraded polysaccharides to scavenge DPPH radicals, superoxide anion radicals, and hydroxyl radicals was greater than that of PP3. In particular, the polysaccharide PP3–4 (20 kDa), which has the smallest molecular weight, showed the highest antioxidant activity [29]. In addition, using RSM, Li et al. determined the ideal parameters for the degradation of *Porphyra haitanensis* polysaccharide with pectinase: 50 U/mL pectinase, a temperature of 47.5 °C, and a pH of 5.0. In this scenario, CPH (Mw = 524 kDa) was degraded into DCPH (Mw = 217 kDa). DCPH demonstrated greater efficacy than CPH in decreasing malondialdehyde and reactive oxygen species production in RAW264.7 cells exposed to hydrogen peroxide, as indicated by in vitro studies. Moreover, DCPH showed the potential to boost the growth, phagocytosis, and nitric oxide secretion of RAW264.7 cells [17]. In summary, numerous studies have shown that the antioxidant activity [29,53], anti-allergic activity [18] and immune regulation ability [7,17] of *P. haitanensis* polysaccharides improve after degradation, with the activity increasing as the molecular weight of the degraded polysaccharide decreases. Degradation can improve some of the biological activities of polysaccharides from *P. haitanensis*, which is undoubtedly an effective means to realize the full utilization and high-value development of polysaccharides from *P. haitanensis* and to develop new ideas and increase the potential application value of polysaccharides from *P. haitanensis* in food and medicine.

### 5.2. Sulfation Modification

**Figure 3:**
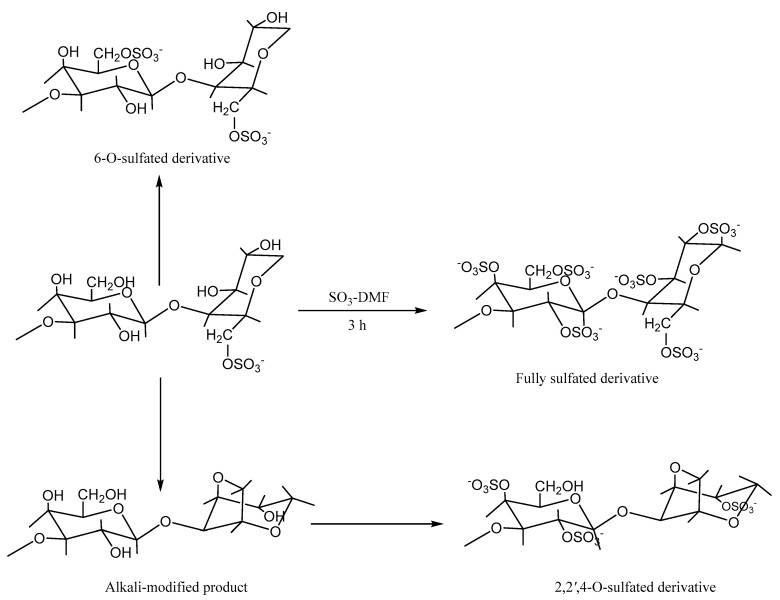
Structure and synthesis of disaccharide units and their derivatives of *P. haitanensis* polysaccharides [70].

Sulfation is a commonly used polysaccharide modification method. Polysaccharides have been shown to have excellent biological activity after sulfation, and studies have shown that sulfate derivatives exhibit good antioxidant, anticoagulant, anti-tumor, antibacterial, and immunological activities [65,66,67]. The sulfation of *P. haitanensis* polysaccharides was studied as early as 2009. For example, Zhang et al. used the sulfated reagent SO_3_-DMF produced from chlorosulfonic acid (HClSO_3_) and N,N-dimethylformamide (DMF) to sulfate polysaccharides from *P. haitanensis* [68]. FT-IR analysis revealed that the sulfated polysaccharides had two characteristic absorption peaks, which were located at 817 cm^−1^ and 1225 cm^−1^, indicating that the sulfate group was connected to the primary hydroxyl group. In addition, compared with that of the original polysaccharide, the S=O vibration of the modified polysaccharide at 1249 cm^−1^ was increasingly wider, and the sulfate content of the modified polysaccharide increased from 15.6% to 27.6%. The above data indicated that the sulfation of polysaccharides was successful. In addition, the antioxidant activity test results indicated that the ability of sulfated *P. haitanensis* polysaccharides to scavenge superoxide anion radicals and hydroxyl radicals was greater than that of the original polysaccharides, which showed stronger antioxidant activity [68]. Furthermore, the biological activity of sulfated polysaccharides is influenced by factors such as the level of substitution, position of substitution, molecular weight, and spatial conformation [69]. Zhang et al. used 4,4′-dimethoxytrityl chloride (DMT-Cl) to replace the hydroxyl groups at different positions on the disaccharide unit in *P. haitanensis* polysaccharides, introduced a DMT protective group, sulfated and modified it with SO_3_-DMF reagent, and deprotected it to obtain fully sulfated porphyrans, i.e., 2,2′,4-O-sulfated derivatives and 6-O-sulfated derivatives [70]. The specific disaccharide unit structure is shown in Figure 3. The results showed that the sulfate contents of the total sulfated derivatives, 6-O-sulfated derivatives, and 2,2′,4-O-sulfated derivatives were 39.20%, 31.43%, and 22.72%, respectively. Among them, the completely sulfated and 6-O-sulfated derivatives exhibited a superior ability to scavenge superoxide anion radicals compared to the original polysaccharides and 2,2′,4-O-sulfated derivatives. Interestingly, the reducing power of the fully sulfated product with a higher degree of substitution was much weaker than that of the 6-O-sulfation product. Obviously, the sulfation of *P. haitanensis* polysaccharides at the O-6 position seems to increase their reducing power. The anticoagulant activity test results confirmed that the anticoagulant activity of completely sulfated derivatives significantly increased, while the anticoagulant activity of 6-O-sulfate derivatives was lower than that of 2,2′,4-O--sulfate derivatives, indicating that the sulfate group on C-6 was not necessary for anticoagulant activity [70]. The above conclusions indicate that the biological activity of sulfated *P. haitanensis* polysaccharide derivatives depends not only on the degree of sulfation but also on the substitution position of the sulfate group. In summary, sulfation improves the anticoagulant and antioxidant characteristics of *P. haitanensis* polysaccharides, broadening the scope of their development and utilization. However, in general, there are few studies on the sulfation of *P. haitanensis* polysaccharides, the modification methods are relatively simple, and the structure–activity relationships of polysaccharides are not clear. Future research on the sulfation of *P. haitanensis* polysaccharides is expected to improve our understanding of the correlation between the chemical structure and biological effects of sulfated *P. haitanensis* polysaccharides, to develop new *P. haitanensis* polysaccharide products and expand the scope of their market application.

### 5.3. 5-Fluorouracil Polysaccharide

**Figure 4:**
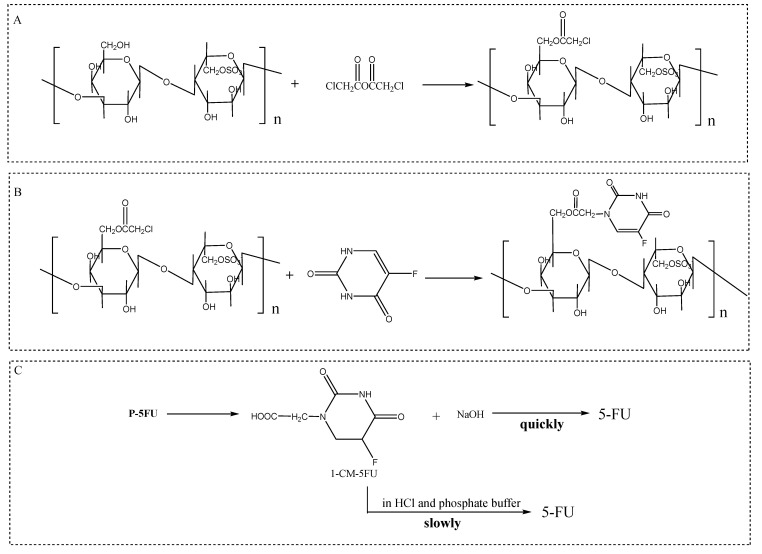
Synthesis and degradation mechanism of 5-fluorouracil polysaccharide. (**A**) Preparation of chloracetylated P; (**B**) preparation of conjugates carrying 5-FU; (**C**) hydrolytic mechanism of conjugates in different mediums [77].

5-Fluorouracil (5-FU) is a first-line chemotherapeutic drug with a wide range of activities against various types of cancers, and it can be used to treat solid tumors such as breast cancer, colorectal cancer, and head and neck cancer [71]. Nevertheless, its anticancer effects are often accompanied by adverse side effects such as reproductive toxicity [72] and cardiac toxicity [73]. Currently, studies have shown that the combination of 5-fluorouracil and natural plant polysaccharides can effectively alleviate and treat the serious side effects caused by the anticancer drug 5-fluorouracil [74,75,76]. In 2009, Zhang et al. conducted a chloroacetylation reaction between *P. haitanensis* polysaccharide P and chloroacetic anhydride under the catalysis of DMAP, and then, the product after chloroacetylation was substituted with 5-fluorouracil to obtain the conjugate P-5FU (the reaction equation is shown in Figure 4). A release mechanism study revealed that this 5-fluorouracil conjugate drug exhibited typical Fickian diffusion [77]. Moreover, Zhang et al. fixed the 6th position of the unit structure of low-molecular-weight polysaccharide (LP) with 5-FU to obtain LP-5FU and then treated different groups of S180 tumor mice with 5-FU and LP-5FU in in vivo experiments. LP-5FU significantly reversed the decrease in the lymphocyte proliferation rate and TNF-*α* and NO levels induced by 5-FU, indicating that the conjugate can increase the anti-tumor activity of 5-FU and improve the immune function disrupted by 5-FU [78]. Therefore, we speculate that *P. haitanensis* polysaccharides are good adjuvants for the treatment of cancer and that 5-FU can also be used as a new potential anticancer drug with low toxicity and side effects.

### 5.4. Other Modification Methods

Phosphorylation and acetylation are also common modification methods of polysaccharides from *P. haitanensis*. Phosphorylation is a frequently utilized chemical modification technique that can improve the physical and chemical properties of natural polysaccharides, thus enhancing their anti-tumor, antioxidant, and other biological activities [79]. *P. haitanensis* polysaccharides have been dissolved in formamide and treated with tributylamine and polyphosphoric acid to obtain phosphorylated *P. haitanensis* polysaccharides [68,80]. FT-IR analysis revealed that phosphorylated *P. haitanensis* polysaccharides have two special absorption peaks: one at 1268 cm^−1^, which represents the stretching vibration related to P=O, and the other at 988 cm^−1^, which represents the vibration related to P–O [80]. The introduction of phosphate groups can activate the hydrogen atoms on the isomer carbon, thereby enhancing the scavenging effect of DPPH free radicals and resulting in good antioxidant capacity [68]. Moreover, acetylation can also effectively improve the biological activity of polysaccharides [81]. For example, *P. haitanensis* polysaccharides are acetylated using acetic anhydride as an acylating agent, with a distinct peak appearing at 1726 cm^−1^ or 1730 cm^−1^ in the infrared spectrum as a result of C=O stretching vibrations [68,82]. Compared with natural *P. haitanensis* polysaccharides, acetylated *P. haitanensis* polysaccharides have a stronger free scavenging ability and reducing energy for superoxide anions [44,58,59]. The reason may be that acetylated derivatives have a greater hydrogen supply capacity, which can prevent the formation of hydrogen peroxide, thus affecting their antioxidant capacity and resulting in stronger antioxidant activity. In addition, drug-loaded polysaccharide metal nanoparticles have also been used as a promising drug delivery system. The principle involves using polysaccharides with an enhanced biological tolerance to encapsulate drug-loaded molecules and load them onto the surface of nanoparticles, so that the conjugates have both a physiological affinity and anti-efflux properties, thereby addressing treatment failure due to the limited delivery of drugs to the target site [83]. Hence, to overcome the poor bioavailability and photostability of resveratrol (RES), Xu et al. prepared OPRs with ovalbumin (OVA) and a *P. haitanensis* polysaccharide (PHP) as encapsulation agents. In vitro simulated digestion experiments showed that compared with OVA-RES nanoparticles (ORs), OPR was released more slowly in the stomach and faster in the intestine. In addition, OPR nanoparticles were shown to inhibit the growth of HeLa and HepG2 tumor cells and effectively deliver Res to biological targets, indicating that the OVA-PHP system may enhance the bioavailability and stability of resveratrol [84].

**Table 3:** Summary of chemical modification methods of *Porphyra haitanensis* polysaccharide.

Classification of Modification	Method	Biological Activity	The Characteristic IR Absorption Peak	References
Degradation	Ascorbate and hydrogen peroxide method	Anti-aging, Antioxidant, and immunostimulatory	N/A	[7,62,63,64]
	Pectinase degradation method	Antioxidant and immunomodulatory		[17,53].
Sulfation	Chlorosulfonic acid and N, N-dimethylformamide method	Antioxidant	New peaks at 1225 and 817 cm^−1^	[68]
	Chlorosulfonic acid and N, N-dimethylformamide method (Regioselective modification of DMT as a protective group)	Antioxidant	New peaks at 1225 and 817 cm^−1^	[70].
		Anticoagulant		
5-fluorouracil polysaccharide	Chloroacetylation and 5-fluorouracil substitution method	Anti-tumor	New peaks at 3428, 2963, 1715–1668 cm^−1^	[77,78]
Acetylation	Acetic anhydride acylation method	Antioxidant	New peaks at 1726 or 1730 cm^−1^	[68,80,82].
Phosphorylation	Tributylamine and polyphosphoric acid method	Antioxidant	New peaks at 1268 and 988 cm^−1^	[68,80]
Drug-loaded nanoparticles	Complex coacervation method.	Anti-tumor	N/A	[84]

N/A means not mentioned.

## 6. Biological Activities of *P. haitanensis* Polysaccharides

**Figure 5:**
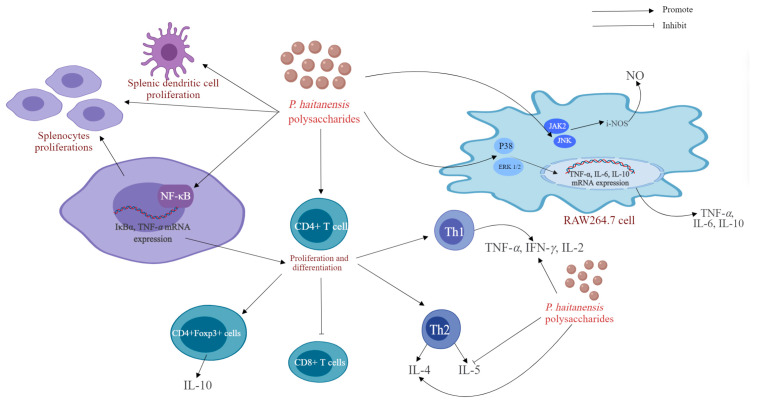
Potential immunomodulatory mechanism of *P. haitanensis* polysaccharides and their derivatives.

**Figure 6:**
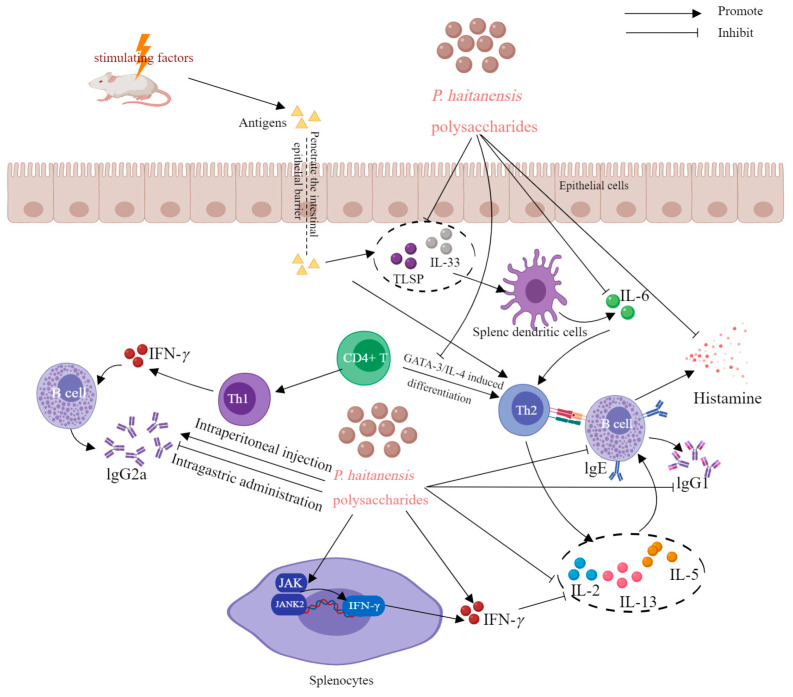
Potential anti-allergic mechanism of *P. haitanensis* polysaccharides and their derivatives.

**Figure 7:**
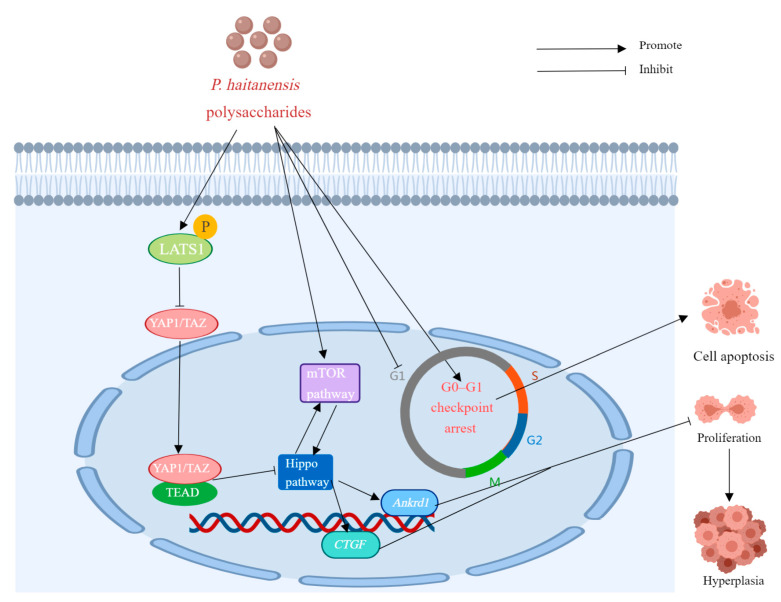
Potential anti-tumor mechanism of *P. haitanensis* polysaccharides and their derivatives.

**Figure 8:**
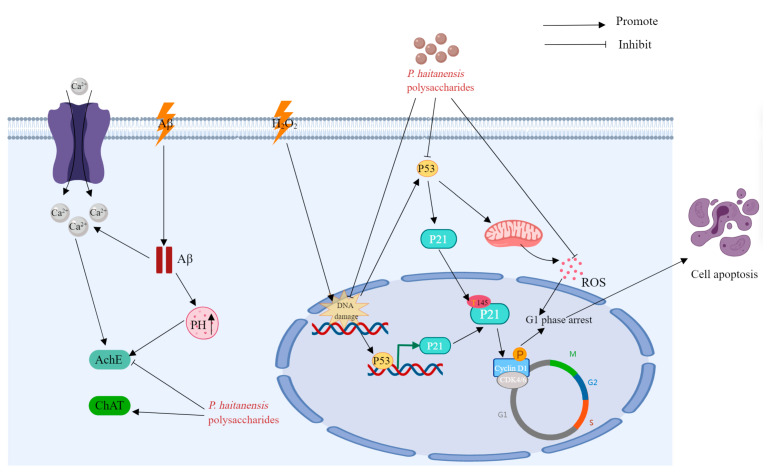
Potential anti-aging mechanism of *P. haitanensis* polysaccharides and their derivatives.

*P. haitanensis* is a kind of medicinal and edible plant from China, and polysaccharide is one of the important bioactive components in *P. haitanensis*. In recent years, numerous studies have validated the diverse biological and pharmacological effects of P. haitanensis polysaccharides, such as antioxidative, immunomodulatory, anti-allergic, anticancer, anti-aging, hypoglycemic, anticoagulant, anticomplement, anti-diarrheal, and other effects. The biological activities and health benefits of P. haitanensis polysaccharides are discussed in the following paragraphs and summarized in Table 4. Typical and representative molecular mechanisms of pharmacological activity—for example, their immunomodulatory, anti-allergic, anti-tumor, and anti-aging activities—are also summarized and presented in Figure 5, Figure 6, Figure 7 and Figure 8.

**Table 4:** Summary of biological activities of polysaccharides from *Porphyra haitanensis*. (↑: improve or promote, ↓: inhibit or reduce.).

Biological Activities	Polysaccharide Names	Types	Testing Subjects	Doses/Duration	Effects/ Mechanisms	Refs.
Antioxidant activity	PHP-KC-AC (HE)	In vitro	DPPH, hydroxyl, and ABTS radicals	0.0625, 0.125, 0.25, 0.5, 1, and 2 mg/mL	At 2.0 mg/mL, the scavenging rates of DPPH, hydroxyl, and ABTS free radicals by PHP were 34.63%, 23.80%, and 53.16%, respectively.	[11]
	PHP3—TZ (HE-DE-SE)	In vitro	DPPH, hydroxyl radical, superoxide anion, and reducing powers	1 to 5 mg/mL	At 5 mg/mL, DPPH and superoxide anion scavenging rates of 52.1% and 56.39%, respectively. At 2 mg/mL, HO• scavenging rates of 22.84%	[14]
	PHPR03 (ME)	In vitro	DPPH, hydroxyl, ABTS, and superoxide anion radicals	2, 4, 6, 8, and 10 mg/mL	Showed the strongest scavenging capability.	[51]
	P3 (D-DE)	In vitro	DPPH, hydroxyl radical and ferric reducing	5 mg/mL	DPPH scavenging and hydroxyl radical rate of, respectively 48.4%, and 38.3%. Reductive ability test solution absorbance is 0.19.	[53]
	PP3–4 (HE-D-DE)	In vitro	DPPH, hydroxyl radical, superoxide anion, and reducing powers	0.5 to 8 mg/mL	Showed the strongest scavenging capability and reducing ability.	[29]
	P3 (HE-D)	In vitro	DPPH radical and ferric reducing	0 to 2.5 mg/mL	Better DPPH radical scavenging activity and reducing ability than original polysaccharide.	[62]
	PHP (HE)	in vitro	DPPH, hydroxyl, ABTS, superoxide radicals, and T-AOC	4 mg/mL	Showed the highest level of hydroxyl radical scavenging ability.	[15]
	PHP0.5–1-UF (HE-DE-SE)	In vitro	DPPH, hydroxyl, ABTS, superoxide radicals, and ferric ion reducing	4 mg/mL	Showed the highest levels of DPPH, superoxide anion radical, and ABTS^+^ radical, as well as T-AOC scavenging ability.	[15]
	P1 P2 (S) P3 (A)	In vitro	DPPH superoxide radicals and ferric ion reducing	0 to 5 mg/mL	The sulfated derivative with certain DS showed stronger antioxidant activity. The acetylated derivative showed the most excellent antioxidant activity.	[82]
	AP (A) PP (P) BP (B)	In vitro	Hydroxyl, superoxide anion radicals, and reducing powers	N/A	The acetylation, phosphorylation, and benzoylation derivatives of *P. haitanensis* polysaccharides showed stronger antioxidant activity than the original polysaccharides.	[80]
	DCPH-TZ-AO (D)	In vitro	Ferric reducing power	1 to 5 mg/mL	The total antioxidant capacity is significant.	[17]
	PHP3—TZ (HE-DE-SE)	In vitro	In H_2_O_2_-stimulated RAW264.7 cells	0, 25, 50, 100, 200, and 400 μg/mL for 1 h	SOD, CAT and GSH-Px ↑; MDA and ROS level ↓;	[14]
	DCPH-TZ-AO (D)	In vitro	In H_2_O_2_-treated RAW264.7 cells	0, 25, 50, 100, 200, and 400 μg/mL for 1 h	MDA and ROS↓; CAT ↑;	[17]
	F1 (HE-S)	In vivo	Aging Kunming mice (20 months old, 35–45 g)	50, 100, and 200 mg/kg/d, i.p., for 20 days	SOD, GSH-Px ↑; MDA ↓;	[56]
Immunomodulating activity	PHPD-IV-4 (HE-D-SE)	In vitro	RAW264.7 cells	0~200 µg/mL for 24 h	Phagocytic uptake, NO, ERK1/2, JNK, and P38, ↑;	[7]
	DCPH-TZ-AO (D)	In vitro	RAW264.7 cells	25, 50, 100, 200, and 400 μg/mL for 24 h	Phagocytic uptake, NO ↑;	[17]
	PHPS (HE)	In vitro	RAW264.7 cells	5, 20, 40, 80 and 100 μg/mL for 12 h or 24 h,	Phagocytic uptake, NO, (IL)-6, IL-10, TNF-α, JNK and JAK2 ↑;	[16]
	PHPS (HE)	In vivo	BALB/c mice	50, 150, and 250 mg/kg/d for i.g.,14 days	Lymphocytes proliferation, splenocytes proliferations TNF-α, IL-10, CD4^+^ Th cells, DCs, and Tregs ↑; CD8^+^ T cells ↓;	[16]
	PH (HE)	In vivo	BALB/c mice	5 mg/d i.g., for three days a week, four weeks in total	NF-κB, IFN-*γ*, TNF-*α*, IL-4 and IL-10 and CD4^+^CD25^+^ Tregs ↑; IL-5 ↓;	[10]
Anti-allergy activity	PHPS (HE-DE)	In vitro	In tropomyosin-sensitized splenic lymphocytes		IL-4, IL-5 and IL-13↓; Th1, IFN-*γ*, IL-10, JNK and JNK2↑;	[19]
	PHPS (HE-DE)	In vivo	In tropomyosin-sensitized mice	100 μg/mouse, i.p.,	IgE, IgG1 ↓; IgG2a ↑;	[19]
	PHPS (HE-DE)	In vivo	In tropomyosin-sensitized mice	100 μg/mouse, i.g	Histamine levels ↓;	[19]
	PP (HE)	In vivo	In OVA-sensitized mice	25, 150, or 250 mg/kg/d, i.g., for 31 days	IgE, IgG1, IL-2, IL-4, and IL-17 ↓; IL-10 ↑;	[85]
	DPHSP (HE-D)	In vivo	In OVA-induced food allergy mouse	50, 150, or 250 mg/kg/d, i.g., for 13 days	IgE, IL-4, IL-13 ↓; Differentiation of Treg cells↑;	[18]
Anti-tumor activity	PHP (ME)	In vitro	Human gastric carcinoma SGC-7901 cells	10, 20, 100, 200, 500 μg/mL for 48 h	Within the concentration range of 10 to 500 μg/mL, inhibition rates increased from 8.25% to 70.40%.	[20]
	PHPs (UME-DE)	In vitro	Human colon cancer HT-29, LoVo, and SW-480 cells	0, 300 and 600 μg/mL for 72 h	Cell hyperplasia and cell G0–G1 phase ↓;	[21]
	PH (HE-DE-SE)	In vitro	Mouse colon cancer CMT93 cells	0.2, 0.4, or 0.8 mg/mL for 24, 48, or 72 h.	Cell hyperplasia, TAZ, YAP1, CTGF and Ankrd1 ↓; LATS1 ↑;	[22]
	PHP (ME)	In vivo	SGC-7901 tumor-bearing mice	40, 80, and 160 mg/kg/d, i.g., for 25 days	Inhibition rates of high-, middle-, and low-dose groups were 57.26%, 67.55% and 77.04%, respectively	[20]
Anti-aging activity	PHP (HE)	In vitro	In H_2_O_2_-treated WI-38 cells	10 g/mL for 2 h	p53-p21 pathways and SAHF-like foci ↓; SA-β-gal positive cells decreases from 53% to 23% in the cultures at 30 PDs.	[24]
	DP (HE-D)	In vivo	Kunming male mice (Aβ_1−40_ induction)	75, 150 and 300 mg/kg/d for 16 days	Cerebral acetylcholine and ChAT ↑; AchE ↓;	[64]
	P (1,2,3) (HE-D)	In vivo	*Drosophila melanogaster*	0.01 to 2 mg/mL	P1 and P2 on 1% diet significantly increased mean lifespan by 8.60% and 6.68%, respectively. For P, flies kept on 0.2% diet significantly increased mean life span by 6.10%.	[23]
Prebiotic activity	PHP-KC (HE)	In vitro	Fermentation of healthy human feces	100 mg/9 mL	*Bacteroides thetaiotaomicron*, *Bacteroides ovatus*, *Defluviitalea saccharophila*, and *Faecalibacterium prausnitzii* ↑; Putrefying bacteria ↓;	[30]
	PHP (UME)	In vitro	Fermentation of healthy human feces	100 mg	*Coprococcus_3, Bacteroides, Sutterella*, *Lachnospiraceae_UCG_006, and Bacteroidales_S24_7_group* ↑; *Escherichia_Shigella and Dorea* ↓;	[37]
	PHP1 (HE-DE-SE)	In vitro	10-week-old SPF rats fecal material (10 g)	10.00 g/L for 24 h	*Escherichia-Shigella* ↓; Propionate-producing, *Ruminococcaceae*_NK4A214, the *norank*_*f*_*Ruminococcaceae*, *Christensenellaceae*_R-7, *Fusicatenibacter*, *Ruminiclostridium*_5, *Blautia, E.coprostanoligenes, Desulfovibrio, Lactobacillus* and *Parasutterella* ↑;	[52]
	PHP2-BZ (HE-DE-SE)	In vitro	Sprague Dawley rat fecal material (~10 g)	10 g/L 24 h	Ruminococcaceae_UCG-005, *Ruminococcus*_2, *Lactobacillus* and *Escherichia-Shigella* ↑;	[57]
Hypolipidemic activity	APHP (HE)	In vivo	In alloxan-induced diabetic mice	100, 200 and 400 mg/kg/d, i.g., for 21 days	TC, TG and LDL ↓; HDL ↑;	[42]
	PHP (HE)	In vivo	In diet-induced high-fat *Mesocricetus auratus*	100, 200 mg/kg/d for 4 weeks	TC, TG, LDLC, Abhd5, Me1, Elovl6, Fasn, and Pnpla3 ↓; Muribaculaceae, Faecalibaculum, CD36, Acacb, and PPARg ↑;	[86]
Hypoglycemic activity	APHP (HE)	In vivo	In alloxan-induced diabetic mice	100, 200, and 400 mg/kg/d, i.g., for 21 days	TC, TG, and LDL ↓; β-cell proliferation and HDL ↑;	[42]
Anticoagulant activity	Sulfated porphyrans	In vitro	Citrated normal chicken plasma	1 to 20 μg/mL	APTT, TT, and PT ↑;	[70]
Anti-complement activity	LP-G2 (HE-D-DE-SE)	In vitro	0.5% rabbit erythrocytes	0 to 3.5 mg/mL 0 to 10 mg/mL	Block hemolysis of SRBC; Selectively interact with C1q, C2, C4, and C9; Classical approach and alternative approach ↓;	[12]
Anti-diarrhea activity	PHSP_(hp)_ (HE)	In vivo	In ETEC-K88 infected mice	10 mg/kg/d, i.g., for 1 week	TNF-*α*, IL-6 ↑; Nitroblue tetrazolium, B cells↓	[58]

The meaning in parentheses is the name: HE: hot water extraction, ME: microwave-assisted extraction, UME: ultrasonic–microwave-assisted extraction, DE: purification by DEAE cellulose column chromatography, SE: purification by gel column chromatography, D: degradation derivatives, S: sulfated derivatives, A: acetylated derivatives, P: phosphorylated derivatives, B: benzoylated derivatives.

### 6.1. Antioxidant Activity

Oxidative stress refers to an imbalance between oxidation and antioxidant systems in the body [87]. The results of recent studies suggest that oxidative stress may lead to numerous severe illnesses, including chronic obstructive pulmonary disease, atherosclerosis, cancer, and Alzheimer’s disease [88]. As biomacromolecules with good biocompatibility [89], natural polysaccharides, which are widely used in the biomedical and pharmaceutical fields, have significant antioxidant effects and can reduce oxidative stress damage [90]. In biological systems, the inappropriate production of endogenous and exogenous reactive oxygen species (ROS) leads to conformational and oxidative changes in key biomolecules, resulting in oxidative damage to cells [91]. Khan et al. reported that a *P. haitanensis* polysaccharide (PHP-KC-AC) was most effective at scavenging ABTS free radicals, followed by DPPH radicals and hydroxyl radicals, and that the antioxidant ability of PHP-KC-AC increased in a dose-dependent manner. As the concentration of PHP-KC-AC increased from 0.0625 mg/mL to 2.0 mg/mL, the scavenging rates of ABTS, DPPH, and hydroxyl radicals increased gradually. Moreover, this dose dependence was more obvious at high concentrations, and the highest activity clearance rates at 2.0 mg/m L were 34.63%, 23.80%, and 53.16% for ABTS, DPPH, and hydroxyl radicals, respectively [11]. Subsequently, Wu et al. isolated PHP using cellulose DEAE-52 ion exchange and Sephadex G-100 column chromatography, yielding three sulfated polysaccharide fractions (PHP, PHP2, and PHP3). Through an assessment of antioxidant activity in vitro, it was found that the three components showed strong superoxide anion free radical, hydroxyl radical, and DPPH free radical scavenging ability and reducing ability; in general, the order of activity was PHP3 > PHP2 > PHP1, and this order was also the order of sulfate content, indicating that the antioxidant activity of the three components was positively correlated with the sulfate content. In addition, in RAW264.7 cells treated with H_2_O_2_, the ability of PHP3 to reduce intracellular ROS levels was the strongest among the three components, and at a dose of 400 μg/mL, the ROS level decreased by 182.37% compared with the level observed in normal cells. Similarly, PHP3 can also reduce the ROS level and MDA content in H_2_O_2_-treated RAW264.7 cells and enhance the activities of catalase (CAT), glutathione peroxidase (GSH-Px), and superoxide dismutase (SOD), thus resulting in better antioxidant activity [14]. Furthermore, a degraded *P. haitanensis* polysaccharide (DCPH, Mw = 217 kDa) showed stronger free radical scavenging activity and iron ion reduction ability than did another *P. haitanensis* polysaccharide (CPH, Mw = 524 kDa). CPH and DCPH decreased the levels of propylene glycol and ROS inside RAW264.7 cells treated with H_2_O_2_, and this effect was dependent on the dose. DCPH exhibited a more potent effect than CPH. The reason may be that the degradation process exposes more hydroxyl groups on DCPH, and that low-molecular-weight DCPH is more conducive to penetrating the cell membrane, thereby facilitating interactions with intracellular substances [17].

In addition, through animal experiments, Zhang et al. found that *P. haitanensis* polysaccharides also showed significant antioxidant activity in vivo. After the intragastric administration of 50, 100, and 200 mg/kg *P. haitanensis* (F1) polysaccharides for 28 days, the activities of SOD in the serum, liver, heart, and brain of aged Kunming mice increased in a dose-dependent manner. Compared with that in the aging model group, the level of GSH-Px in the administration group was also obviously increased. The dose that resulted in the greatest increase in GSH-Px activity in the liver and brain was 5.0 mg/kg, and the greatest increase in GSH-Px activity in the heart was observed at a dose of 100 mg/kg. At doses of 100 and 200 mg/kg, F1 evidently inhibited the production of MDA in the liver and heart of aging mice, and the inhibitory effect of a 200 mg/kg dose of F1 on MDA was comparable to or stronger than that of the same dose of vitamin C (a positive control drug) [56]. In conclusion, *P. haitanensis* polysaccharides have good antioxidant effects, are a good natural antioxidant, and can be used for nutritional and health food development and utilization. However, there are few studies on the in vivo antioxidant activity of polysaccharides from *P. haitanensis*, and the mechanism of in vivo activity is not clear. Therefore, it is necessary to further carry out scientific research on antioxidation in vivo, and fully analyze and summarize the antioxidation effects of polysaccharides from *P. haitanensis*, in vitro and in vivo, so as to provide a more valuable theoretical basis for the clinical application of *P. haitanensis* polysaccharides.

### 6.2. Immunomodulatory Activity

The body’s first line of defense against threats is the immune system; it can not only identify hosts and pathogens, to eliminate infection and maintain the body’s dynamic balance, but it can also protect the body from bacteria, fungi, viruses, and other infections and eliminate abnormal cells such as tumor cells through cellular and humoral immunity [92,93]. Research has shown that natural plant polysaccharides have good immunomodulatory effects and are good immune enhancers. Polysaccharides can attach to receptors on the surface of cells, which can increase engulfment by macrophages and stimulate the release of cytokines and chemokines by macrophages [94,95]. *P. haitanensis* polysaccharides can also facilitate the proliferation of immune cells and the production of cytokines by immune cells, thereby improving the immune response and enhancing the body’s immune system. In recent years, in vivo and in vitro studies have shown that *P. haitanensis* polysaccharides have good immunomodulatory effects; their possible effects and mechanisms of action are shown in Figure 5.

Macrophages, a crucial component of the immune system, serve as the initial barrier against both internal and external pathogens [96], regulating a variety of dynamic balances and evolutionary host defense immune responses [97]. In vitro experiments have shown that *P. haitanensis* polysaccharides can greatly improve the survival rate of RAW 264.7 cells, increase phagocytic activity in a dose-dependent manner, and stimulate the release of TNF-*α*, IL-6, and IL-10. Moreover, *P. haitanensis* polysaccharides activate the JNK and JAK2 signaling pathways in RAW 264.7 cells, induce increased iNOS mRNA expression, and promote NO production [16]. Similarly, Gong et al. treated RAW 264.7 cells with different concentrations of purified polysaccharide (PHPD-IV-4) from 12.5 μg/mL to 200 μg/mL. Their findings indicated notable increases in the phagocytic capacity, intracellular acid phosphatase activity, and NO generation of RAW 264.7 cells, and the phagocytic activity (phagocytosis index = 1.57), acid phosphatase activity (vitality index = 1.37), and NO production ability (28.67 μg/mL) were greatest when the concentration of PHPD-IV-4 was 200 μg/mL. Furthermore, Western blot results demonstrated that PHPD-IV-4 notably increased the phosphorylation levels of three components of the MAPK signaling pathway, ERK1/2, P38, and JNK, in a dose-dependent fashion, suggesting that PHPD-IV-4 modulates macrophage immune activity by enhancing the phosphorylation of MAPK signaling molecules [7].

Dendritic cells (DCs) are antigen-presenting cells that are crucial for initiating and controlling both innate and adaptive immune reactions [98]. CD4+ regulatory T cells are important for maintaining immune self-tolerance and a dynamic balance [99]. Liu et al. studied the immunomodulatory activity of *P. haitanensis* sulfated polysaccharides (PHSPs) in BALB/c mouse models. According to the flow cytometry results, PHSPs enhanced the generation of TNF-α and IL-10 in mice, with CD4+ spleen T lymphocytes, DCs, and Tregs showing notable increases in mice treated with PHSPs [16]. Combined with the experimental results of Liu et al., these findings confirmed that PHSPs play a key role in immune regulation. Subsequently, Fu et al. reported that a *P. haitanensis* polysaccharide (PH) can activate the NF-κB signaling pathway in mouse spleen cells, thereby inducing Treg proliferation. In addition, PH may promote the production and release of Th1 cytokines (IFN-*γ*, TNF-*α* and IL-2) and promote and inhibit the secretion and expression of the Th2 cytokines IL-4 and IL-5, respectively. This demonstrates that PH has the ability to control the equilibrium between Th1 and Th2 cells by regulating the secretion and gene expression of Th1 and Th2 cytokines, thus maintaining a dynamic immune balance [10].

In summary, the immunomodulatory effect of polysaccharides from *P. haitanensis* is mainly achieved by regulating the activity of immune cells such as lymphocytes, macrophages, and DCs and promoting or inhibiting the expression of immune factors to maintain the Th1/Th2 balance. This indicates that *P. haitanensis* polysaccharides can be used as immunomodulators or immune supplements in food and medicine to treat immune diseases. However, the available studies on the immunomodulatory effects of *P. haitanensis* are insufficient, and there is a lack of research and discussion on the mechanisms of action involved. Therefore, it is necessary to further study the immunomodulatory effects and mechanisms of action of *P. haitanensis* polysaccharides, especially in vivo, to establish a strong theoretical foundation for their development and application.

### 6.3. Anti-Allergy Activity

Food allergy is an immune system response that occurs shortly after eating certain foods, affecting more than 10% of the global population and causing severe harm to individuals with allergies [100]. Studies have shown that natural polysaccharides have a good ability to mitigate food allergies and can be used as potential products for preventing and treating food allergies [101]. Shi et al. studied the anti-allergic ability of *P. haitanensis* sulfated polysaccharide (PHSPs) in a tropomyosin (TM)-induced model of allergy in mice. Mice were sensitized through the intraperitoneal administration of TM (100 μg/mouse) at 0 and 14 days. The sensitized mice received intraperitoneal injections of PHSPs three times weekly for 15 days, starting either 7 days prior to sensitization (PHSP prevention group) or 1 day after the initial immunization (PHSP treatment group). These findings indicated that PHSPs significantly decreased the expression of Th2 cytokines (including IL-4, IL-5, and IL-13), while enhancing the release of Th1 cytokines (such as IFN-*γ* and IL-10). This regulatory change affected the levels of various specific immunoglobulins in serum. PHSP intervention before TM sensitization significantly decreased the level of TM-specific immunoglobulin IgE in serum and increased the level of TM-specific IgG2a. After TM sensitization, there was no significant change in TM-specific IgE or IgG2a in the serum after PHSP treatment, but the TM-specific IgG1 level was significantly decreased. In addition, further studies have shown that PHSPs can activate the JNK and JAK2 pathways to induce IFN-*γ* secretion [19]. These results strongly suggest that PHSPs prevent or ameliorate TM-induced allergic reactions by regulating the Th1/Th2 immune imbalance. Moreover, Wei et al. studied the antigen-specific immunity of a *P. haitanensis* polysaccharide (PP) in ovalbumin (OVA)-sensitized mice. Mice received injections of 40 and 80 μg/mouse OVA on days 4 and 8, respectively, and were treated with 25, 150, or 250 mg/kg PP daily for 30 days. PP treatment decreased the levels of the two immunoglobulin subclasses, IgG1 and IgG2a, which assist Th2 and Th1 immune responses, and PP significantly decreased the concentration of the OVA-specific immune protein IgE at doses of 150 and 250 mg/kg. Additionally, PP inhibited the production of Th17 cytokines (IL-17) and Th2 cytokines (IL-4 and IL-2) and inhibited and promoted the expression of the Th1 cytokines IFN-*γ* and IL-10b, respectively, leading to a notable increase in the IL-10/IL-4 ratio [85]. In summary, PP can shift the Th1/Th2 balance to Th1 polarization, thereby regulating antigen-specific immune responses and reducing allergic reactions. In addition, research was conducted on the anti-allergic properties of degraded polysaccharides from *P. haitanensis* and polysaccharides from *P. haitanensis* harvested at various times. The lower the molecular weight and sulfate content were, the better the anti-allergic activity was [13,18].

In conclusion, *P. haitanensis* polysaccharides can inhibit allergic reactions by regulating Th1/Th2 immune imbalances. We summarize various possible relationships involved in this regulatory process in Figure 6. However, the regulatory effect of *P. haitanensis* polysaccharides on allergic reactions is complex and changeable. For example, the effects of *P. haitanensis* polysaccharides on Th1 cytokines (IFN-γ) and the immunoglobulin subclass IgG2a are different in different environments. For example, mice were treated with an intraperitoneal injection of *P. haitanensis* polysaccharides, which promoted the secretion of IFN-*γ* and IgG2a, while Wei et al. found that the intragastric administration of *P. haitanensis* polysaccharides reduced the levels of IFN-γ and IgG2. The reason for the differences may be that the different routes of administration affect the absorption, distribution, and metabolism of polysaccharides in the body, which in turn alters their effects. In addition, the application range of *P. haitanensis* polysaccharides is different, and their mechanisms of action are also different. As shown above, the effects of *P. haitanensis* polysaccharides on immunoglobulin in allergic reactions are also different. Therefore, future studies should further clarify the anti-allergic mechanism of *P. haitanensis* polysaccharides and explore the effects of different administration routes or drug dosage forms on their activity, to provide a theoretical basis for the development of anti-allergic health foods and drugs containing *P. haitanensis* polysaccharides and to broaden the research scope of *P. haitanensis* polysaccharides.

### 6.4. Anti-Tumor Activity

Cancer is a highly dangerous disease that has become the most serious threat to human life and health, solidifying it as a major public health problem worldwide [102]. Polysaccharides are bioactive macromolecules that are considered potential anticancer candidates because of their anti-tumor activity and nontoxic properties [103]. Seaweed polysaccharides have been shown to be important active substances in seaweed and to display anticancer activity [104,105]. Polysaccharides of *P. haitanensis*, a food homologous to traditional Chinese medicines derived from algae, show potential as candidate drugs for cancer treatment due to their anticancer activity. Chen et al. studied a polysaccharide (PHP) extracted from *P. haitanensis* by MAE and found that it had obvious anti-tumor effects in vitro and in vivo. The suppressive impact of PHP on the proliferation of human gastric carcinoma SGC-7901 cells was confirmed through in vitro experimentation. PHP was found to suppress the growth of SGC-7901 cells in a dose-dependent manner. With an increasing PHP concentration (10–500 μg/mL), the inhibition rate also gradually increased, and the inhibition rate of cell proliferation increased from 8.25% to 70.40%. In addition, PHP induced apoptosis in SGC-7901 cells. At 500 μg/mL, the percentage of apoptotic cells was 19.65%, and the percentage of apoptotic cells was positively correlated with the PHP concentration. In vivo experiments showed that PHP had a strong anticancer impact on mice with SGC-7901 tumors. At doses of 80 and 160 mg/kg/d, the tumor inhibition rates were 67.55% and 77.04%, respectively, which were better than that of the positive control drug 5-Fu (58.3157%) [20]. Furthermore, Yao et al. isolated and purified three components (PHP-F1, PHP-F2, and PHP-F3) from the polysaccharides of *P. haitanensis*. In vitro studies revealed that the three components inhibited the proliferation of human colon cancer cells (HT-29, LoVo, and SW-480), with PHP-F2 and PHP-F3 demonstrating particularly potent inhibitory effects on HT-29 cells. Further studies revealed that PHP-F2 and PHP-F3 can effectively trigger oxidative stress in HT-29 cells and promote apoptosis. Moreover, PHP-F2 and PHP-F3 can arrest cells in the G0–G1 phase and prolong cell cycle progression, thereby inhibiting cell division and ultimately leading to apoptosis [21]. Moreover, a *P. haitanensis* polysaccharide (PH) exhibited a notable suppressive effect on CMT93 colon cancer cells. PH inhibited the expression of the core transcription coactivator and regulator *YAP1* in a dose-dependent manner, activated the Hippo pathway, caused crosstalk between the Hippo and mTOR pathways, and activated the mTOR pathway, thereby inhibiting the expression of the *CTGF* and *Ankrd1* genes and thus inhibiting the proliferation of intestinal epithelial cells [22].

Based on the above findings, we summarized and analyzed the possible anti-tumor mechanism of *P. haitanensis* polysaccharides, as shown in Figure 7. *P. haitanensis* polysaccharides can achieve anti-tumor effects by interfering with the cell cycle and the Hippo/mTOR pathway. However, the existing anti-tumor research on *P. haitanensis* polysaccharides is still relatively basic, and there is a lack of in vivo experimental studies. The immune system is crucial for monitoring cancer cells [93], and *P. haitanensis* polysaccharides are good immunomodulatory substances. Whether the immunomodulatory effect of *P. haitanensis* in vivo affects its anti-tumor activity remains to be determined. Therefore, the relationship between the immune activity and anti-tumor activity of polysaccharides from *P. haitanensis* should be further explored in future studies. We hope to develop a product that can inhibit tumor growth without side effects, while enhancing the body’s immune system.

### 6.5. Anti-Aging Activity

Aging is considered one of the greatest known risk factors for morbidity and mortality. As early as 2008, some scholars found that polysaccharides from *P. haitanensis* could effectively enhance the vitality of middle-aged *Drosophila melanogaster* and prolong its life span. In particular, polysaccharides with lower molecular weights may be more conducive to increasing the growth of *Drosophila melanogaster* [23]. The oxidative stress theory is considered a famous aging mechanism theory. Mitochondrial ROS are thought to significantly contribute to the aging process, as oxidative stress-induced mitochondrial dysfunction is recognized as a key factor in aging [106]. In addition, the aging of the brain is linked to oxidative damage to nuclear and mitochondrial DNA, which involves the regulation of various systems by reactive oxygen species (ROS) through different mechanisms [107]. The effect of *P. haitanensis* polysaccharides on premature senescence induced by H_2_O_2_ in a WI-38 model was studied in vitro. *P. haitanensis* polysaccharides had obvious growth-promoting effects on cells. At a polysaccharide concentration of 10 μg/mL, the viability of prematurely senescent WI-38 cells increased from 70.3% to 88.5%; additionally, *P. haitanensis* polysaccharides significantly restored the destruction of cell morphology caused by H_2_O_2_. Moreover, polysaccharides from *P. haitanensis* suppressed the activation of the p53 and p21 proteins and decreased ROS production in cells, thus preventing oxidative stress and minimizing DNA damage in aging cells [24]. In recent years, it has been recognized that aging is a sign of the aging of the nervous system, manifesting as memory loss and a deterioration in mental state [108]. Zhang et al. reported that the degradation of a *P. haitanensis* polysaccharide (DPPH) improved the cognitive function and memory deficits caused by neurotoxic amyloid β peptide (Aβ) in mice with Alzheimer’s disease (AD). AD mice were continuously treated with DPPH (75, 150, 300 mg/kg) for 16 days. The results from the water maze test indicated that the number of times the mice entered the maze and the time it took for them to find the exit on the third day were notably shorter in the 300 mg/kg group than in the control group (*p* < 0.05). Furthermore, the number of errors and time to exit decreased significantly in all the dose groups after the fifth day (*p* < 0.01). In addition, further studies have shown that DPPH can increase acetyltransferase (CHAT) activity in the cerebral cortex and hippocampus, and reduce acetylcholinesterase (AChE) activity and increase acetylcholine content in brain tissue, thereby improving Aβ1–40-induced learning and memory impairment in mice [64] (the possible detailed mechanism of action is shown in Figure 8). The above results indicate that *P. haitanensis* polysaccharides can delay aging and improve memory, suggesting that they can be developed as a new type of anti-aging health food to prevent neurodegenerative diseases.

### 6.6. Prebiotic Activity

In recent years, there has been increased focus on the impact of the gastrointestinal microflora on human health and diseases. The human gastrointestinal microbiota is a highly diverse microbial population, whose composition and diversity are associated with a variety of pathologies [109]. Moreover, immune cells and symbiotic microorganisms in the human intestinal tract constantly communicate and react with each other in a stable environment to maintain healthy immune activities [110]. Recent studies have indicated that natural active polysaccharides can regulate gastrointestinal function, contribute to the growth of probiotics in the intestine, and inhibit the formation of harmful bacteria in the intestine. Through in vitro fermentation experiments with intestinal microorganisms, Ying et al. fermented a polysaccharide from *P. haitanensis* (PHP) in the feces of healthy adults and found that PHP significantly promoted the growth of beneficial bacteria such as *Bacteroides thetaiotaomicron, Bacteroides ovatus, Defluviitalea saccharophila*, and *Faecalibacterium prausnitzii*; additionally, this selective influence on the intestinal flora led to an increase in the diversity of the human gut microbiota [30]. Chen et al. isolated and purified two polysaccharide components (PHP1 and PHP2) from PHP and then examined the fermentation of PHP1 and PHP2 in rat intestinal microbiota in vitro. PHP1 enhanced the development of bacteria that produce propionic acid and the synthesis of propionic acid; promoted the growth of *Ruminococcaceae*_NK4A214, *Christensenellaceae*_R-7, *Fusicatenibacter*, *Ruminiclostridium*_5, *Blautia*, *E. coprostanoligenes*, *Desulfovibrio*, *Lactobacillus,* and *Parasutterella*; regulated microbial diversity; and increased the abundance of beneficial microflora. In addition, the molecular weight of PHP1 decreased from 6.68 × 10^6^ g/mol to 5.46 × 10^5^ g/mol during fermentation. The reason was that the increase in *Bacteroides*, *Ruminococcaceae*, *Bifidobacterium,* and other genera caused the cleavage of glycosidic bonds in PHP1. For example, *Bifidobacteria* glycosyl hydrolases can hydrolyze the *α*- or *β*-galactose glycosidic bond in PHP1 [52]. Additionally, through metabolic studies, it was found that the microbial flora consumed galactose and mannose in PHP2 during in vitro fermentation, resulting in less metabolism of microbial fructose, mannose, and galactose. PHP2 also reduced amino acid metabolism and carbohydrate metabolism in the gut microbiota. In addition, PHP2 inhibited the biosynthesis of secondary bile acids and primary bile acids in the microflora and reduced the level of intestinal bile acids. Moreover, PHP2 regulated the composition of the fecal microbiota in rats and greatly increased the abundance of the dominant genera *Escherichia-Shigella* and *Lactobacillus*. The analysis showed that a decrease in bile acid levels could promote the conversion of cholesterol to bile acid in the liver, suggesting that PHP2 regulates cholesterol metabolism [57]. The above results show that *P. haitanensis* polysaccharides have great potential for development into new probiotic products for use in functional foods or by the pharmaceutical industry. However, research on the effect of *P. haitanensis* polysaccharides on the intestinal flora is limited to in vitro experiments. More studies on the effect of *P. haitanensis* polysaccharides on intestinal flora in in vivo treatments are needed to increase the efficacy of *P. haitanensis* polysaccharides in improving or regulating microbial flora.

### 6.7. Other Activities

In addition to the above biological activities, *P. haitanensis* polysaccharides also have other pharmacological effects, including hypoglycemic, hypolipidemic, anti-complement, anti-diarrheal, and anticoagulation effects. For example, *Mesocricetus auratus* fed a high-fat diet was also fed PHP at doses of 100 mg and 200 mg/kg/d for 4 weeks. PHP in both dose groups significantly suppressed an increase in serum total cholesterol (TC), total triglyceride (TG), and low-density lipoprotein cholesterol (LDL) levels. PHP (100 mg/kg) downregulated the expression of a fat chain extension gene (Elovl6), a fatty acid synthesis gene (FASN and Pnpla3), a fat degradation gene (Abhd5), and an adipogenesis gene (ME1), thereby reducing triglyceride and blood lipid levels. PHP (200 mg/kg) also inhibited the expression of a fatty acid oxidation gene (Acacb) and fatty acid transporter gene (CD36) by activating the liver PPARg signaling pathway, thereby reducing liver triglyceride and blood lipid levels [86]. Additionally, Cao et al. reported that a polysaccharide (APHP) extracted from waste *P. haitanensis* stimulated the proliferation of β-cells in a mouse model of diabetes induced by alloxan, promoted the regeneration of damaged islet cells, and significantly reduced blood glucose levels. Additionally, APHP improved antioxidant activity, reduced ROS levels, reduced the degree of damage to β-cells, reduced LDL, TG, and TC levels, increased high-density lipoprotein (HDL) cholesterol levels, and restored blood lipid levels [42]. These results indicate that *P. haitanensis* polysaccharides have good hypoglycemic and hypolipidemic activities and can potentially be used as functional foods for the treatment of diabetes and its complications.

Moreover, in vitro anticoagulation experiments showed that sulfated *P. haitanensis* polysaccharides could inhibit the endogenous coagulation pathway, thrombin-mediated fibrin formation pathway, and exogenous coagulation pathway to achieve anticoagulant effects [70]. Zhang et al. reported that degraded sulfated heterogalactan (LP-G2) can inhibit the hemolysis of sheep red blood cells (CH50 = 3.08 ± 0.25 mg/mL) through the classical pathway and can also inhibit the hemolysis of rabbit red blood cells (AP50 = 2.23 ± 0.20 mg/mL) through the alternative pathway. Further studies have shown that the anti-complement bioactivity of LP-G2 is reflected in its ability to exert anti-complement effects through the above two pathways and that LP-G2 selectively interacts with C1q, C2, C4, and C9 [12].

Furthermore, in BALB/c mice infected with the enterotoxin *Escherichia coli* (ETEC-K88), the rate of infectious diarrhea decreased within the first four days following the oral intake of *P. haitanensis* polysaccharide (PHSP_(hp)_) at a concentration of 10 mg/kg, with all affected mice recovering to normal by the fourth day. Compared with the weight loss of the mice in the diarrhea group after 5 consecutive days, the weight loss of the mice in the PHSP_(hp)_ treatment group rebounded on the second day, suggesting that PHSP_(hp)_ could reduce the diarrhea rate and increase body weight, thus relieving diarrhea symptoms. Additionally, PHSP_(hp)_ has been shown to decrease nitroblue tetrazolium levels in mouse serum and decrease the number of B cells, thereby suppressing the production of proinflammatory cytokines and immunoglobulin A secretion and reducing the inflammatory response to diarrhea induced by ETEC-K88 through specific and nonspecific immunity [58].

In summary, *P. haitanensis* polysaccharides show rich biological activity and have an important practical significance, warranting further exploration and application. However, the relationship between the unique structure and structure–activity relationship of the polysaccharides of *P. haitanensis* and their biological activity needs to be further elucidated, which would provide a favorable basis for promoting the creation of health products and clinical therapeutic drugs containing *P. haitanensis* polysaccharides.

## 7. Conclusions and Future Prospects

As early as in ancient China, *P. haitanensis* was shown to have good medicinal value and has been an important food source for coastal residents. Polysaccharides, as important active components of *P. haitanensis*, have important research value. Studies have confirmed that the polysaccharides of *P. haitanensis* display rich biological activity, revealing that the polysaccharides of *P. haitanensis* have considerable development and utilization value. In recent years, research on *P. haitanensis* has focused mainly on extraction, purification, modification, structural identification, and biological activity. The above aspects have been reviewed and discussed in detail, but some conclusions and viewpoints need to be further elaborated.

First, the methods for extracting polysaccharides from *P. haitanensis* mainly include the HWE method, MAE method, and UMAE method, but at present, the extraction methods are relatively basic, with low extraction rates. In the future, in the development of products from *P. haitanensis* polysaccharides and in putting *P. haitanensis* polysaccharides into industrial production, the extraction and purification yield of *P. haitanensis* polysaccharides will be problematic, because their yield will directly affect the economic benefits of the product. Therefore, it is necessary to explore and innovate high-yield, environmentally friendly, economical, and practical extraction and purification process routes, while standardizing the extraction and purification of *P. haitanensis* polysaccharides.

Second, the monosaccharide composition, monosaccharide sequence, molecular weight and type, and location of glycosidic bonds are the main focus of structural analyses of *P. haitanensis* polysaccharides. *P. haitanensis* polysaccharides generally contain more sulfate (3.7–14.7%), and the main monosaccharide is Gal. The main linkage mode of *P. haitanensis* polysaccharides is *β*- or *α*-glycosidic bonds, and the basic structural skeleton is 3) *β*-D-galactose (1→4), 3,6-anhydro-*α*-L-galactose (1→, and→3), *β*-D-galactose (1→4), *α*-L-galactose-6-S (1→). However, their detailed structural composition and conformational characteristics, especially the relationship between the structure and biological activity of *P. haitanensis* polysaccharides and their derivatives, have not been fully studied and need to be further explored. In the future, it will be necessary to use more advanced analytical techniques, such as X-ray diffraction, SEM, AFM, capillary electrophoresis and Raman spectroscopy, to analyze the advanced structure of *P. haitanensis* polysaccharides. In addition, the molecular modification of polysaccharides from *P. haitanensis* can change the structural characteristics and biological functions of polysaccharides. For example, the introduction of sulfate groups could enhance the anticoagulant and antioxidant activities of *P. haitanensis* polysaccharides, and different substitution degrees and substitution sites alter the biological activity and activity intensity of *P. haitanensis* polysaccharides. *P. haitanensis* polysaccharides can also be used as drug carriers. The combination of *P. haitanensis* polysaccharides and 5-FU can alleviate the toxicity and side effects of 5-FU, and the introduction of *P. haitanensis* polysaccharides into the OVA-RES nanoparticle system can improve the stability and bioavailability of resveratrol. Moreover, the basic structure of *P. haitanensis* polysaccharides does not change after degradation and modification, but their antioxidant activity, immune activity regulation, and anti-allergic activity improve. The reason may be that degraded small molecules are more conducive to absorption by the body. Therefore, we speculate that the basic skeleton of *P. haitanensis* polysaccharides is likely one of the key factors determining their activity; therefore, further studies on the composition of the basic skeleton of *P. haitanensis* or its modification are likely to reveal additional biological activities. However, the substitution site and mechanism of action involved in the modification process of *P. haitanensis* polysaccharides are still unclear. In particular, the impacts of the addition of new functional groups on chemical structure and conformational characteristics, as well as the degree of derivatization and safety, need to be further clarified.

Third, as a biomolecule, the molecular structure of *P. haitanensis* polysaccharides is highly diverse and complex; therefore, it is very important to study their advanced structural characteristics and the relationships of those characteristics with biological activity. Furthermore, *P. haitanensis* polysaccharides, as a traditional Chinese food and medicine, could be studied and identified by querying ancient records and using intestinal flora analysis, metabolomics, proteomics, genomics, and other technologies to investigate and discover additional pharmacological activities of *P. haitanensis* polysaccharides. Overall, there will be many opportunities and challenges in future research on polysaccharides from *P. haitanensis*. Researchers can reveal the diverse biological activities of *P. haitanensis* polysaccharides in medicine and food and improve the understanding of the pharmacological mechanism and clinical applications of polysaccharides from *P. haitanensis*. Overall, research on extraction and purification processes, on the chemical modification of *P. haitanensis* polysaccharides, and on the chemical synthesis of high-value drugs based on the biological activities of *P. haitanensis* polysaccharides has shown great promise.

## Data Availability

Not applicable.

## Abbreviations

*P. haitanensis*	*Porphyra haitanensis*
HWE	Hot water extraction
MAE	Microwave-assisted extraction
UMAE	Ultrasonic–microwave-assisted extraction
HPSEC	High-performance size exclusion chromatography
HPGPC	High-performance gel permeation chromatography
GC-MS	Gas chromatography-mass spectrometry
HPLC	High-performance liquid chromatography
FT-IR	Fourier transform infrared spectroscopy
NMR	Nuclear magnetic resonance
UV	Ultraviolet analysis
Gal	Galactose
Glu	Glucose
Man	Mannose
Ara	Arabinose
Fuc	Fucose
Xyl	Xylose
Rha	Rhamnose
Rib	Ribose
GalN	Galactosamine
GalUA	Galacturonic acid
GlcN	Glucosamine
GulUA	Guluronic
ManUA	Mannuronic
GlcUA	Glucuronic acid
3,6-AG	3,6-anhydro galactose
H_2_O_2_-VC	Hydrogen peroxide–Vitamin C
DMF	N,N-dimethylformamide
5-FU	5-Fluorouracil
AFM	Atomic force microscopy
SEM	Scanning electron microscopy
ROS	Reactive oxygen species
